# Unveiling the potential of alginate-based nanomaterials in sensing technology and smart delivery applications

**DOI:** 10.3762/bjnano.15.88

**Published:** 2024-08-22

**Authors:** Shakhzodjon Uzokboev, Khojimukhammad Akhmadbekov, Ra’no Nuritdinova, Salah M Tawfik, Yong-Ill Lee

**Affiliations:** 1 Department of Pharmaceutical Sciences, Pharmaceutical Technical University, Tashkent 100084, Republic of Uzbekistan; 2 Department of Petrochemicals, Egyptian Petroleum Research Institute (EPRI), Nasr City, Cairo 11727, Egypt; 3 Anastro Laboratory, Institute of Basic Science, Changwon National University, Changwon 51140, Republic of Korea

**Keywords:** alginate, biomedical sensing, polymer nanoparticle, smart drug delivery

## Abstract

Sensors are applied to many fields nowadays because of their high sensitivity, low cost, time-saving, user-friendly, and excellent selectivity. Current biomedical and pharmaceutical science has one focus on developing nanoparticle-based sensors, especially biopolymeric nanoparticles. Alginate is a widely used biopolymer in a variety of applications. The hydrogel-forming characteristic, the chemical structure with hydroxy and carboxylate moieties, biocompatibility, biodegradability, and water solubility of alginate have expanded opportunities in material and biomedical sciences. Recently, research on alginate-based nanoparticles and their applications has begun. These materials are gaining popularity because of their wide usage potential in the biomedical and pharmaceutical fields. Many review papers describe applications of alginate in the drug delivery field. The current study covers the structural and physicochemical properties of alginate-based nanoparticles. The prospective applications of alginate-based nanomaterials in various domains are discussed, including drug delivery and environmental sensing applications for humidity, heavy metals, and hydrogen peroxide. Moreover, biomedical sensing applications of alginate-based nanoparticles regarding various analytes such as glucose, cancer cells, pharmaceutical drugs, and human motion will also be reviewed in this paper. Future research scopes highlight existing challenges and solutions.

## Review

### Introduction

Sensors have received a lot of attention in a variety of applications, including health, pharmacy, the environment, and industry [1–7]. When employing these technical devices, users can chose user-friendly, cost-effective, sensitive, and error-free detecting instruments [8]. The field of biosensors has received a lot of interest over the last years because of their amazing capacity for early disease diagnosis, quick detection of diverse drugs and chemicals, and long-term monitoring [9]. In the recent decade, sensor technology has seen breakthroughs thanks to the usage of nanomaterials with superior physicochemical properties [10–13]. Nowadays, the development of sensors based on nanomaterials, particularly alginate-based, has raised the interest of many in the biomedical field for monitoring and regulating human health [14].

Biopolymers are naturally occurring polymeric compounds derived from living organisms [15–18]. They are mostly used in pharmaceutical and biomedical applications. Biodegradable and bioabsorbable polymers are an excellent choice for a variety of innovative drug delivery systems (DDSs). Biopolymers are also used in cutting-edge scientific applications such as gene expression, tissue engineering, smart drug delivery, and biosensors [11,19]. Modern medicine and pharmacy are looking for such materials for DDSs because targeted drug delivery is becoming a vital way of carrying drugs. Traditional drug delivery methods including tablets, capsules, syrups, and ointments, have significant disadvantages such as low bioavailability and variability of drug levels in plasma, which makes them incapable of continuous release [20]. In addition, to achieve optimal efficacy and safety, the drug must be administered at a precisely controlled rate and a special target site [21]. To solve these issues in DDSs, biopolymers can be a perfect solution. Because they have several excellent properties that can be crucial in drug delivery, such as low cost, antioxidant and antibacterial activity, non-toxicity, biodegradability, and biocompatibility. Biopolymeric nanoparticles (NPs) can be used in DDSs, and they can protect the drugs from the adverse conditions of the gastrointestinal tract to deliver the active pharmaceutical substance without any damage to its site of action. In addition to that, biopolymers can protect patients from the negative effects of chemicals, serving as a small container to safely store drugs inside. Moreover, biopolymers are not harmful to the human body, as they are antimicrobial, biodegradable, and non-toxic [22].

Biopolymeric nanoparticles are a very effective material for producing biosensors. In today’s world, people need sensors to monitor various types of pollution. Food contamination with infectious microorganisms or air and water contamination with heavy metals are good examples of the need for sensors [23]. In addition, incurable diseases such as cancer can be detected by biosensors, and the application of biosensors is also very important for medicine and pharmaceuticals [18,24–25]. Natural biopolymers are abundant and exhibit variable chemical compositions, customizable characteristics, easy processability, great biocompatibility and biodegradability, and nontoxicity, opening up new avenues for the creation of flexible sensing technologies [26–27]. Alginate-based nanomaterials are among the most widely studied biopolymers for drug delivery and sensing applications because of their properties.

**Figure 1:**
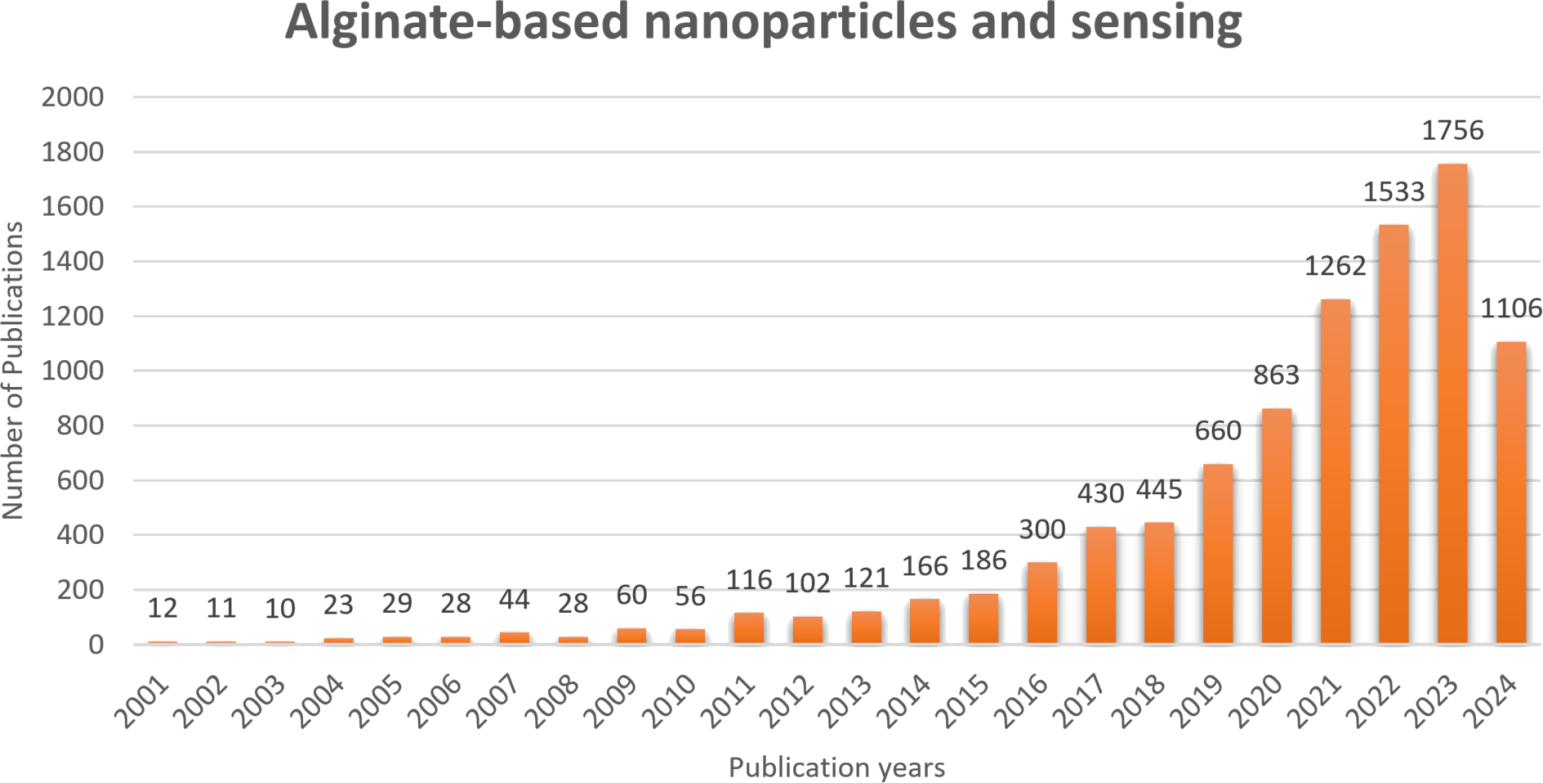
The number of publications of alginate-based nanoparticles in sensing techniques over the years. Keywords: alginate-based nanoparticles and sensing (Source: https://www.sciencedirect.com).

Sodium alginate is a biopolymer from the sea, and it is one of the most commonly utilized natural materials in several pharmaceutical applications such as smart delivery systems and sensors [11,19]. The chemical structure of alginate indicates that it belongs to the hydrogel family and is insoluble in water. Sodium alginate is an odorless, tasteless powder that can be white or yellowish. Alginate is a linear polymer composed of ᴅ-mannuronic acid (M) and ʟ-guluronic acid (G) residues [28]. Alginate can be an effective absorbent and homeostatic dressing agent for wound healing. In addition, it can be utilized as an emulsifier and thickener in cosmetics, dentistry, and tissue engineering [29]. Furthermore, alginate decreases stomach inflammation and helps the healing of the gastric mucosa. Consequently, it can both protect the stomach and ease discomfort. Gastric dressing occurs when sodium alginate and hydrochloric acid are combined in the stomach. Additionally, alginate can prevent obesity by lowering body weight [30]. All the qualities make sodium alginate indispensable for a wide range of applications in medicine and sensing. As a result, several researchers are working on alginate to create nanoparticles. One of the most appealing properties of alginate is its capacity to readily form a gel with divalent cations, particularly Ca^2+^ [31]. Divalent cations create hydrogen bonds with the alginate G residues, resulting in an egg-box gel structure. As a result, the viscosity and other mechanical qualities of alginate can be enhanced, allowing for the formation of a hydrogel. The attractive properties of alginate include low cost, abundance, biocompatibility, biodegradability, antibacterial activity, non-toxicity, and the ease to form gels. Sodium alginate, especially, sodium alginate hydrogel is becoming more attractive for sensing applications. This hydrogel can be used for tackling severe water contaminations with heavy metals and food contaminations [32]. Nanoparticles of the hydrogel have gained attention regarding sensing applications in recent years (Figure 1). Figure 1 shows the increasing number of publications on alginate-based nanoparticles in sensing applications.

**Figure 2:**
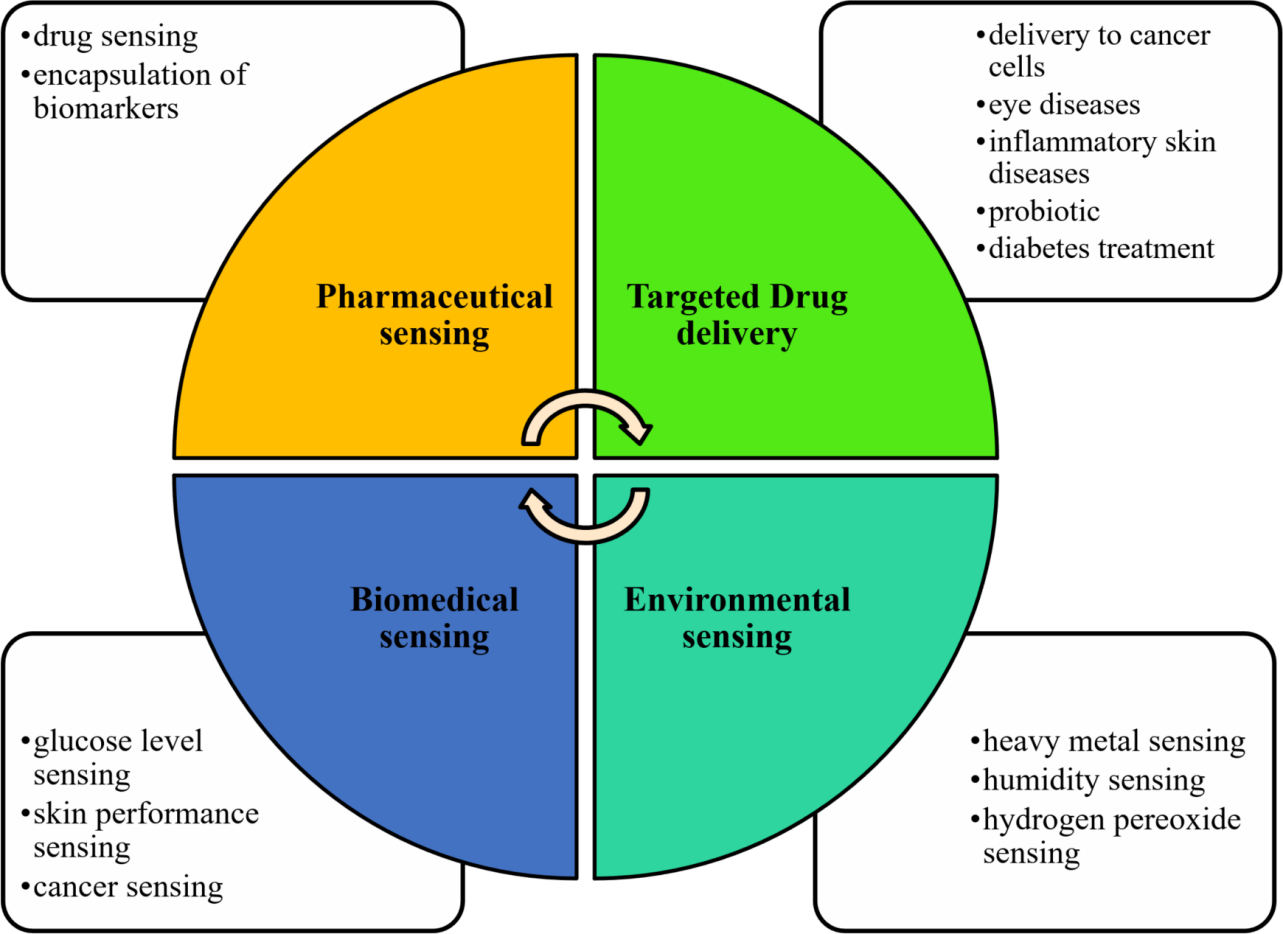
Applications of alginate-based nanomaterials in sensing and smart drug delivery.

Many review articles have been written so far to describe drug delivery applications of sodium alginate. Niculescu et al. reviewed recent applications of alginate-based nanoparticles for biomedical applications and the food industry [33]. Another study on alginate-based nanoparticles described the synthesis and characterizations of nanoparticles and their applications to drug delivery, tissue engineering, and gene therapy [34]. Other recent review papers have also emphasized alginate-based nanoparticles for drug delivery, wound healing, and controlled release of drugs [35–37]. There are few studies that reviewed alginate-based materials for sensing, pharmacy, and biomedicine. Therefore, this review article is based on recent research on alginate-based composite nanoparticles, which provides a comprehensive overview of nanoparticles and their uses, especially in sensing applications (Figure 2). Moreover, characterization techniques of biopolymeric nanoparticles and their importance for drug delivery and sensing methods will be reviewed. In addition, the advantages of alginate-based materials over other drug delivery methods and enhancement for sensing applications will be compared. Furthermore, several environmental, biomedical, and pharmaceutical sensing applications of alginate-based nanocomposites will be reviewed. This review will also discuss the current challenges in the drug delivery field and sensing techniques and the significant role of alginate-based nanoparticles in overcoming these problems.

### Background

#### Drug delivery and sensing in healthcare: significance and impact

Drug delivery refers to the process of administering therapeutic molecules, such as medications, to patients in a targeted and controlled manner. The main goal is that the drug should be delivered to its intended site of action, at the right time and concentration, and achieve maximum therapeutic efficacy with minimal side effects [38]. This is important because traditional drug delivery methods often result in suboptimal drug concentrations at the target site, leading to inadequate treatment outcomes or unnecessary side effects.

One of the key implications of drug delivery is its potential to enhance the effectiveness and safety of drug therapies. For example, it is possible to deliver medications to cancer cells with little side effects and minimum damage to healthy cells. As a result, DDSs can improve patient compliance and adherence to medications [39]. Furthermore, smart drug delivery can also increase the bioavailability of drugs, which refers to the proportion of the administered drug that enters the target systemic circulation. Another important aspect of drug delivery is its ability to overcome biological barriers and provide the availability in drug-specific body tissues. For example, in the case of Alzheimer’s or Parkinson’s disease, drug delivery methods that can effectively cross the blood–brain barrier are essential for delivering therapeutic agents to the brain [40]. Nanotechnology has emerged as a promising tool in smart delivery systems. Nanoparticles can be designed to pass through biological barriers and reach specific sites in the body, increasing the delivery efficiency of drugs [41].

In addition to drug delivery, sensing technologies are also of great importance in healthcare. Sensing technologies enable healthcare professionals to monitor patients’ health conditions, track the effectiveness of treatments, and detect any potential risks or complications [42]. Recently, researchers and medical organizations have begun using low-cost biosensors to monitor food and water toxins, human biological processes, accurate health diagnostics, and for other applications [43]. Biosensor-based technologies are essential to assess samples precisely in modern medical equipment [44]. Biosensors are now transitioning from laboratory-based systems to distributed health care. Traditional scientific diagnosis involves taking a sample from the patient and sending it to an analytical laboratory. In these point-of-care tests, the patient has to wait several hours for results. The development of compact and portable miniaturized sensors has enabled the detection of different biomarkers for continuous, real-time human health monitoring [1–2]. Temperature, heart rate, electrical conductivity, dehydration, and glucose are some of the indicators that sensors can analyze.

#### Challenges in traditional drug delivery methods

Traditional methods of drug administration only deliver non-targeted drugs to the human body without any protection. As a result, the chemical substance can have a negative effect not only on the site of drug action but also on other parts of the human body. Despite several advantages, such as simplicity of administration and patient acceptance, conventional DDSs have significant limitations and disadvantages. They have limited efficacy through varying absorption rates of the drugs when given orally. Also, a low-pH environment and digestive enzymes in the gastrointestinal tract can break down some medications before they reach the bloodstream [45]. In addition to this, lack of selectivity can minimize their effectiveness. Also, drug absorption may be high in detoxifying organs such as liver and kidneys, causing toxicity in those organs [46]. Another drawback is that current drug delivery technologies yield only limited bioavailability and change drug levels in plasma, making them incapable of long-term release. Without proper dispensing techniques, the entire treatment process may fail. In addition, the drug must be delivered at a precisely regulated rate and location to ensure optimal efficacy and safety [21]. Medicines can become the cause of disease when considering the disadvantages such as instability, uncontrollable discharge, irritation and pain, poor absorption, and enzymatic degeneration. However, if nanoparticles, especially biopolymer nanoparticles, are applied to drug delivery methods, the aforementioned drawbacks faced by traditional drug delivery today can be overcome.

#### The evolution of nanoparticle-based drug delivery techniques

Drug delivery via nanoparticles has transformed the world of medicine in recent decades. These techniques aim to improve disease treatment by increasing targeted accumulation of drugs into diseased tissues while minimizing side effects and toxicity. Nanoparticle-based drug delivery strategies have evolved since the 1970s, when researchers first investigated the use of nanoparticles as drug carriers. Since then, tremendous progress has been achieved in the design, manufacture, and characterization of nanoparticle-based DDSs. The use of alginate in drug delivery goes back to the 1980s, when researchers first investigated its ability to encapsulate pharmaceuticals. Over time, researchers have improved the formulation procedures for alginate-based nanoparticles to improve drug-loading capacity, stability, and controlled release characteristics. To improve the formulation of alginate nanoparticles, several methods have been developed, including emulsion-based approaches, solvent evaporation, and nanoprecipitation. Advances in alginate-based nanoparticle design have resulted in increased drug encapsulation efficiency, allowing for larger drug payloads within the nanoparticles. Biopolymeric nanoparticles have become the most commonly used nanoparticle DDSs in recent years.

#### The advantages of using biopolymeric nanoparticles

Nanoparticle systems offer several advantages in drug delivery [41,47]. One of the major advantages of nanoparticle-based DDSs is that they can protect drugs and ensure the delivery of drugs to targeted cells or tissue [48]. By encapsulating drugs within nanoparticles, the delivery system can specifically target the diseased tissue, ensuring that the highest concentration of the drug reaches the desired location while minimizing systemic exposure. This targeted delivery leads to enhanced therapeutic outcomes and reduced side effects [49]. Another advantage of biopolymeric nanoparticles in drug delivery is the ability to improve oral bioavailability. Nanoparticles made from biopolymers can enhance the absorption rate and stability of orally administered drugs, allowing for improved treatment efficacy of medicines [50]. Furthermore, biopolymeric nanoparticles can sustain the medicine or gene effect in targeted tissues. This sustained release of medication extends the duration of the effect of the drug, ensuring optimal treatment efficacy. Additionally, biopolymeric nanoparticles can carry various functional groups on their surface enabling targeted drug delivery. These functional groups can include ligands or antibodies that specifically bind to the target cells or tissues [51]. Stimuli-responsive nanoparticles help reduce toxicity and control drug biodistribution [46].

### Alginate-based nanoparticles

#### The preparation methods of alginate-based nanoparticles

The preparation of alginate-based nanoparticles involves a series of steps that utilize green chemistry principles [52]. Green chemistry involves using sustainable and environmentally friendly processes to minimize the use of hazardous materials and reduce waste. To begin the preparation of alginate-based nanoparticles, a nontoxic metal ion is chosen as the precursor. This precursor can be combined with alginate, a natural polymer derived from seaweed, in a purified natural solvent such as water. Then, a reducing agent is selected to reduce the metal ions and form nanoparticles. One environmentally friendly option for reducing the metal ions and forming nanoparticles is to use sodium alginate as both a capping and reducing agent [53–55]. The synthesis of alginate-based nanoparticles involves the following steps: First, alginate polymer is dissolved in water. Next, the metal salt is added to the alginate solution, and the nanoparticle formation reaction is initiated by adjusting the pH. During the reaction, sodium alginate acts as a reducing agent, as it contains carboxylic groups that can interact with the divalent cations and reduce them to form nanoparticles. After the formation of metal nuclei, these nuclei grow and accumulate within the alginate matrix, resulting in the formation of alginate-based nanoparticles. The morphology and size of the nanoparticles can be controlled by adjusting various specifications of materials such as the concentration of alginate, metal ion precursor, pH, and reaction time. When it comes to the alginate–polymer combination, there are various methods available for the preparation of alginate-based biopolymeric nanoparticles, each with its advantages and limitations. One common method is the ionotropic gelation method, which involves the cross-linking of alginate and polymer in the presence of divalent ions such as calcium or zinc. The microemulsion protocol is another technique that can be used to prepare alginate nanoparticles. The hydrogel formation method is yet another method for preparing alginate–polymer nanoparticles [56]. Another method for preparing alginate–polymer nanoparticles involves the use of oil-in-water emulsions in polymer and calcium alginate solutions to prepare polymer NPs and calcium alginate NPs separately [57].

#### Characterization techniques of alginate-based nanoparticles

Characterization techniques play a crucial role in analyzing biopolymeric nanoparticles, providing valuable information about their physical and chemical properties. These techniques allow researchers to understand the structure, stability, surface properties, and drug release behavior of biopolymeric nanoparticles, enabling them to optimize drug delivery strategies and ensure efficacy and safety [58]. The most crucial characteristics of nanoparticles are particle size, morphology, zeta potential, and surface area.

**Morphology of nanoparticles:** There are many tools available for determining the morphology of nanomaterials. However, the most commonly used methods are scanning electron microscopy (SEM) and transmission electron microscopy (TEM). The shape and size of the nanoparticles can be determined by these two methods [59]. TEM is extensively utilized and can differentiate between nanocapsules and nanospheres, as well as measure the thickness of the nanocapsule wall [60]. Another important morphological feature of polymers is the surface of the polymers, and atomic force microscopy (AFM) can be utilized to detect surface features of polymeric nanoparticles. It is very useful tool that offers high-resolution images in three dimensions at the nanometer scale [61].

**Nanoparticle size:** The nanoparticle size can be determined using a variety of methods including dynamic (DLS) and static (SLS) light scattering; TEM, SEM, and AFM are also widely employed [62–63]. DLS and SLS can detect particle size by determining changes in distribution of particle size, while TEM and SEM yield images of separated particles [61].

**Surface area:** The reactivity of nanoparticles and their ability to interact with ligands highly depend on their surface area. This property of the nanoparticles can be detected directly by adsorbing an inert gas under various pressures to form a monolayer of gas coverage. The surface area of nanomaterials can also be determined by X-ray photoelectron spectroscopy and secondary ion mass spectroscopy [64–65].

**Zeta potential:** The zeta potential of nanoparticles can be calculated from the electrophoretic mobility of particles in a particular solvent using the Doppler approach, which measures particle velocity as a function of voltage. The determination of the zeta potential is crucial in understanding the mechanism of drug–nanoparticle interactions [66].

In addition to the methods described above, Fourier-transform infrared spectroscopy is frequently used to determine the chemical composition and functional groups of biopolymeric nanoparticles [67].

#### Advantages of using alginate-based nanoparticles

The use of biopolymeric nanoparticles, specifically chitosan–alginate nanoparticles, offers several advantages in different fields such as agriculture, medicine, food packaging, and DDSs [68]. One of the advantages of using alginate-based nanoparticles is their biocompatibility. These nanoparticles are made from nontoxic and biodegradable polymers, making them safe to use in various applications [69]. Another advantage is their low cost, as alginate and other polymers are readily available and affordable materials. The combination of alginate and other polymers in nanoparticles results in increased cross-linkage, leading to a denser structure [70]. This denser structure enhances the stability and durability of the nanoparticles, making them suitable for long-term use. Moreover, the alginate-based nanoparticles have demonstrated their suitability for the controlled release of bioactive materials. The nanoparticles can encapsulate and deliver various active compounds, such as polyphenolic compounds, in a controlled manner. Also, these combined nanoparticles have demonstrated enormous advantages for sensing applications. First, alginate-based nanoparticles have a high surface area-to-volume ratio. This feature allows for increased interaction with the target analyte, leading to enhanced sensitivity and detection capabilities. Additionally, alginate-based nanoparticles have excellent biocompatibility, which is crucial for sensing applications in biological systems [71]. Their biocompatible nature ensures minimal interference with the sample or surrounding environment, thereby enabling accurate and reliable measurements. Moreover, alginate-based nanoparticles offer excellent stability and durability, which is essential for long-term sensing applications [72]. Furthermore, these nanoparticles can encapsulate and protect sensitive sensing elements, such as enzymes or receptors, enhancing their stability and preserving their activity over extended periods. Also, alginate-based nanoparticles possess excellent mechanical and electrical properties, overcoming the limitations typically associated with biomaterials in sensing applications [73].

### Drug delivery applications

#### Alginate-based nanoparticles for smart DDSs

Drug delivery systems are one of the areas in which many scientists are working today. One of the leading areas of research is the search for new DDSs and new modes of administration. It includes multidisciplinary scientific approaches to achieve significant progress in increasing the therapeutic index and bioavailability of certain drugs [74]. Conventional drug delivery techniques may be ineffective in many applications. Furthermore, it is difficult to manage the components that are produced in an acidic or basic environments, resulting in a reduction in the therapeutic impact of the medications.

In recent years, smart DDSs have emerged as a promising tool to improve the treatment efficacy of drugs while minimizing adverse effects. These systems involve the use of nanomaterials, such as nanoparticles, to encapsulate and deliver drug substances to specific locations in the body [75]. Alginate-based nanoparticles have gained attention due to their unique characteristics (Table 1), including a surface that easily adheres to the intestinal epithelium, drug encapsulation without the use of organic solvents, good absorption properties, and a low level of toxicity [76]. The adherence to the intestinal epithelium is particularly beneficial for drugs that need to be delivered to the gastrointestinal tract, as it ensures maximum absorption and therapeutic efficacy. Drug encapsulation without organic solvents is advantageous because organic solvents can be toxic and pose a risk to patient safety. The nanoparticles have a large surface area-to-volume ratio, allowing for efficient absorption and transport of drugs. Furthermore, alginate-based nanoparticles are biocompatible and biodegradable, minimizing the risk of long-term side effects.

**Table 1:** Alginate-based nanoparticles and their characteristics for drug delivery.

№	Polymer name	Particle size nm	Zeta potential	Drug loading efficiency	Highlights	Ref.

1.	boronated chitosan/alginate nanoparticles (BCHI/ALG NPs)	296.4 ± 11.2	38.5 ± 2.0	98.1 ± 0.1%	cervical cancer	[77]
2.	capsaicin-loaded alginate nanoparticles (Cap in ALG NPs)	109.5 ± 82.9	−24.9 ± 0.7	98.7 ± 0.6%	prevention and treatment of breast cancer.	[78]
3.	amygdalin-loaded anionic alginate–chitosan nanoparticles	119 ±19	−36.1 ± 0.88	≈90.3 ± 1.5%	delivery to cancer cells	[79]
4.	alginate betamethasone sodium phosphate ALG-BM	155.5 ± 93.2	−24.23 ± 1.2	90%	eye diseases	[80]
5.	alginate-chitosan nanoparticles (CANPs)	≈349.3	≈+29.1	–	antilipidemic formulation	[81]
6.	chitosan oligosaccharide/alginate NPs	264 ± 32	−22.1 ± 1.3	71.3 ± 2.2%	nutraceutical or functional foods	[82]
7.	chitosan–oleic acid-sodium alginate NPs	40–160	45 ± 5	–	therapeutic approach against macular degeneration and diabetic retinopathy	[83]
8.	alginate–chitosan nanoparticles CANPs	300 ± 30	−31.4 ± 0.8	50 ± 1%	*S. aureus* infections	[84]
9.	piperine nanoparticles (NPPi)	104.8 ± 2.3	−25.3 ± 4.20	76.2 ± 0.102%	inflammatory skin diseases	[85]
10.	alginate-based electrospun nanofibers	305	−8 to −9	86%	probiotic	[86]
11.	octaarginine-modified insulin alginate nanoparticles	300	+15	79%	protein drugs	[87]
12.	revaprazan solid lipid nanoparticles (REV-SLNs)	120	−20.7	88%	treating peptic ulcers	[88]
13.	vildagliptin psyllium and alginate nanoparticles VG@P/A-NPs	131 to 650	−22.4	95%	diabetes treatment	[89]
14.	fluorouracil encapsulated alginate nanoparticles 5FU-Alg-Np	254.71 ± 3.98	−38.72 ± 2.91	78.23 ± 3.26%	treatment of local skin cancers	[90]

Boronated chitosan/alginate nanoparticles (BCHI/ALG NPs) were developed and evaluated as a targeted mucoadhesive DDSs for cervical cancer [77]. The BCHI/ALG NPs were less than 390 nm in size and showed a drug encapsulation efficiency of 98.1–99.8%, and a drug loading capacity of 326.9–332.7 g/mg. Surprisingly, boronated chitosan-loaded alginate demonstrated a greater mucoadhesive capacity compared to CHI/ALG NPs, yielding sustained release of the medicine and promising results as a transmucosal DDSs for hydrophobic medicines.

Another enhanced cancer drug delivery system was developed by Iranian scientists. They conceived a nanoparticles-in-nanofibers DDS, which they called nano-in-nano delivery technique, to mitigate limitations of simple nanostructures such as low stability and unsuitable drug release features. They investigated capsaicin-loaded alginate nanoparticles embedded in polycaprolactone–chitosan nanofiber mats. This DDS can extend the release time of capsaicin to more than 500 h compared to the initial 120 h. Furthermore, in vitro tests showed the effective inhibition of MCF-7 human breast cells without any damage to human dermal fibroblasts (HDFs). The created new alginate-based nanoplatform can be used in targeted and prolonged drug delivery of capsaicin in cancer prevention and therapy [78].

Alginate-based fully natural nanoparticles were developed and used for cancer treatment. Pakistani scientists investigated amygdalin in an alginate–chitosan-based matrix [79]. Amygdalin is a natural material and it has very strong anticancerous activity. Scientists achieved over 90% drug encapsulation of amygdalin and sustained drug release for 10 h. The results also showed increased cytotoxicity and controlled release of the drug because of the biodegradable and biocompatible carrier. Alginate–chitosan nanoparticles can be employed as an effective drug delivery vehicle for sustained and controlled amygdalin release, with an increased cytotoxic effect on cancerous cells while sparing normal cells and tissues.

Alginate nanoparticles can be applied to ocular DDSs [91]. Kianersi and coworkers investigated alginate-based nanoparticles for betamethasone sodium phosphate delivery in the human eye [80]. According to the literature, less than 5% of the conventional medication reaches the targeted eye tissue. This emphasizes the need to invent a DDS that stays on the surface of the eye and ensures continuous drug release, increasing the bioavailability of the medication and reducing the need for frequent drug administration. Scientists prepared alginate nanoparticles coated with the two biopolymers chitosan and gelatin. The results demonstrated better encapsulation efficacy (40%) and loading capacity (7%). In addition, in vitro drug release experiments indicated a sustained drug release over 120 h. The longer release time improves patient compliance by reducing administration frequency. Furthermore, continuous drug release can aid in keeping the medication concentration within its therapeutic range. These first findings indicate that the produced alginate nanoparticle-based delivery method has promising potential.

**Figure 3:**
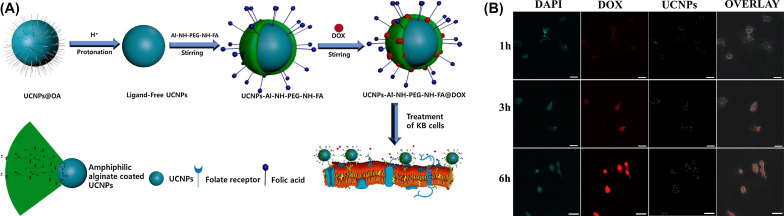
(A) Schemes for preparation of UCNPs capped with the Al-NH-PEG-NH-FA polymer and its applications. (B) Images of KB cells by Laser scanning confocal microscopy, which incubated with DOX-loaded UCNP-Al-NH-PEG-NH-FA (DOX = 42.5 mg·mL^−1^) for 1 h, 3 h and 6 h at 37 °C. DAPI (blue), UCNPs (green) and DOX (red) were recorded in the wavelength ranges of 400–500, 500–700, and 500–600 nm, under 359, 980, and 488 nm laser excitations, respectively. The identical instrumental conditions were used for all illustrations and presented scale bar is 20 mm. Figure 3 was reprinted from [19], Journal of Industrial and Engineering Chemistry, Vol. 57, by S. M. Tawfik, M. Sharipov, B. T. Huy, Z. Gerelkhuu, D. Biechele-Speziale, Y.-I. Lee, “Naturally modified nonionic alginate functionalized upconversion nanoparticles for the highly efficient targeted pH-responsive drug delivery and enhancement of NIR-imaging”, pages 424-435, Copyright (2018), with permission from Elsevier. This content is not subject to CC BY 4.0.

Alginate can be employed also in the form of pH-responsive nanoparticles. pH-responsive amphiphilic alginate can offer hydrophobic drug loading space as well as controlled drug release. As a good example, Tawfik et al. [19] worked on amphiphilic alginate derivatives and conjugated folate (FA) and polyethylene glycol (PEG) onto the alginic acid backbone. These compounds can be utilized in the encapsulation of doxorubicin (DOX) by coating with upconversion nanoparticles (UCNPs), as shown in Figure 3A. When DOX-loaded UCNP-Al-NH-PEG-NH-FA was used, an effective encapsulation efficiency of 81.2% and a high drug loading capacity of 18.3% were obtained. Furthermore, at pH 5.0, 90% of the DOX was released from the conjugated NPs. An acidic environment can be cause for the reduced electrostatic interaction between alginate and DOX. It is noteworthy that KB cancer cells effectively absorbed DOX-loaded UCNP-Al-NH-PEG-NH-FA by FR-mediated endocytosis, which resulted in intracellular pH-triggered DOX release and consequent apoptosis (Figure 3B) [19,47].

#### Chemically modified derivatives of alginate for DDS

Sodium alginate has many carboxylate groups and can be used as a reactive polymer. In the preparation of chemically modified alginate nanoparticles, the dehydration condensation reaction between carboxylate (–COO–) and other chemical groups, such as amino groups, plays a crucial role in forming the cross-links that stabilize the nanoparticles. The procedure generally includes preparing an alginate polymer solution to which a compound with amino groups is subsequently added. In the presence of a cross-linking agent or under appropriate conditions, the carboxylate groups of alginate and the amino groups of the other compound undergo dehydration condensation processes. These cross-links aid in stabilizing the alginate nanoparticles while also controlling their size, shape, and characteristics. These alginate nanoparticles have potential in drug delivery, food technology, and other disciplines that need regulated and focused distribution.

Fenn et al. introduced a methacrylate alginate matrix in which carboxyl groups of alginate were cross-linked with methacrylate and dialdehyde. They used this matrix as a sealant patch for the lungs and for the controlled delivery of soluble drugs such as DOX. The oxidation of alginate produces functional aldehyde groups, which that can form imine bonds with proteins prevalent in many tissues. The controlled release and bioavailability of DOX encapsulated in the alginate matrix enabled the creation of drug-eluting adhesive patches, which were successful in reducing human lung cancer cell (A549) survival [92].

Another research studied modified alginate materials for mucoadhesive drug delivery. Scientists combined the alginate hydroxy groups with maleimid-terminated PEG. This novel mucoadhesive biopolymer exhibited polymer–mucus glycoprotein interactions. The results showed that the new alginate-based mucoadhesive matrix can enhance the prolonged release of drugs as well as the cytotoxicity properties of drugs [93].

Similar research was conducted with starch and alginate for drug delivery applications [94]. Researchers modified the alginate backbone with starch by a green facile technique for controlled drug delivery applications. The authors used theophylline and bovine serum albumin as drug models and alginate–starch polymer as a drug matrix for studying the controlled release. Research confirmed that modification of alginate with starch can improve the encapsulation efficiency of the matrix from 60% to 75%. Moreover, this novel drug carrier protected drugs from the gastric environment and provided the complete release of model drugs in intestinal fluid. In addition, the prepared matrix has zero toxicity and high biocompatibility.

### Sensing applications

#### Alginate-based nanoparticles for sensor technology

Alginate nanoparticles have become known as a promising tool for sensing applications in recent years [95]. With their large surface area, alginate nanoparticles provide space for the immobilization of sensing elements, allowing for enhanced sensitivity and selectivity [96]. Additionally, alginate nanoparticles possess excellent stability and biocompatibility, ensuring that they are eligible for multiple sensing applications without compromising the integrity of the system. Furthermore, alginate is a natural polymer, making it a favorable choice for sensing applications because of its biocompatibility and low toxicity [97]. The application of alginate nanoparticles in sensing is diverse, with notable applications in clinical diagnostics, environmental monitoring, food quality control and processing, pharmaceuticals, and agriculture. One area where alginate nanoparticles have shown significant potential is in the field of clinical diagnostics. Researchers have successfully utilized alginate nanoparticles for the detection and quantification of various analytes, such as proteins, enzymes, nucleic acids, and pathogens. For example, alginate nanoparticles have been used for the detection of cancer biomarkers in body fluids, allowing for early diagnosis and improved treatment outcomes [98]. In environmental monitoring, alginate nanoparticles have been employed for the detection of pollutants and contaminants in water and air, helping to ensure environmental safety [99]. The main factor for the sensing ability of alginate is the surface charge on the polymer. It is critical in enabling quick electron transfer between an enzyme and an electrode surface, triggering the enzyme’s catalytic function for rapid biosensing [100].

#### Environmental sensing applications

One key advantage of using nanosensors in environmental sensing is their ability to detect a wide range of contaminants with high selectivity, accuracy, and sensitivity [101–102]. One of the main advantages of alginate-based nanoparticles is their biocompatibility and biodegradability, which make them ideal for use in environmental sensing. Furthermore, the availability of alginate from marine sources adds to its appeal as a sustainable and renewable material for nanoparticle synthesis [103]. These nanoparticles have demonstrated potential in sensing applications, including temperature, humidity, water level, and various environmental pollutants.

**Heavy metal and volatile organic compounds sensing:** Metal sensing has become a crucial area of research because of the detrimental effects of heavy metal ions on living organisms and ecosystems [104–105]. Metal ions, such as silver, are commonly found in water systems because of industrial activities. In recent years, the use of nanoparticles for metal sensing has gained significant interest among researchers and scientists [106]. Nanoparticles offer several advantages for metal sensing, including high sensitivity, real-time detection, and ease of sample processing [106–108]. Alginate-based nanoparticles have emerged as a promising platform for metal sensing. First, alginate is non-toxic and biocompatible, making it safe for use in various applications [109]. Second, alginate has carboxyl groups, which can capture metallic cations in solutions and achieve local enrichment of the metal ions inside the nanoparticles [110]. This property allows the alginate-based nanoparticles to effectively bind and detect heavy metal ions present in water systems. The synthesis of alginate-based nanoparticles for metal sensing involves the incorporation of metallic nanoparticles, such as silver nanoparticles or zinc oxide nanoparticles, into the alginate polymer network. This can be achieved through various methods, including reduction reactions, electrochemical deposition, or deposition-precipitation. The resulting hybrid nanomaterials exhibit synergistic properties of both the inorganic nanoparticles and the alginate polymer, leading to superior functionality and enhanced metal sensing capabilities [111].

**Figure 4:**
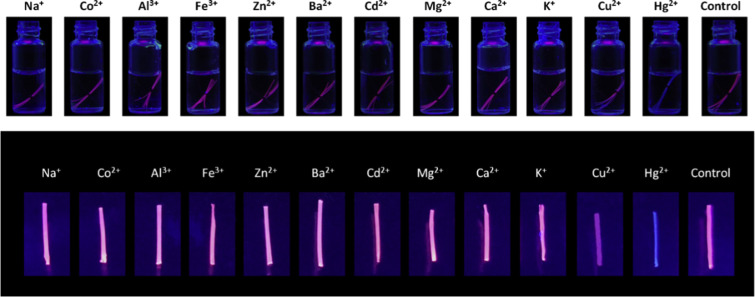
Images of fibers in vials at 1.0 µM concentration of different heavy metal ions. Figure 4 was reprinted from [112], Spectrochimica Acta Part A: Molecular and Biomolecular Spectroscopy, Vol. 230, by Y. He, E. Du, X. Zhou, J. Zhou, Y. He, Y. Ye, J. Wang, B. Tang, X. Wang, “Wet-spinning of fluorescent fibers based on gold nanoclusters-loaded alginate for sensing of heavy metal ions and anti-counterfeiting”, 118031, Copyright (2020), with permission from Elsevier. This content is not subject to CC BY 4.0.

He et al. developed fluorescent fibers from alginate reinforced with gold (Au) nanoclusters by using a wet-spinning technique [112]. The fluorescent fibers exhibited good selectivity and sensitivity towards Cu^2+^ and Hg^2+^. Based on the quenching effect, a design for a “turn-off” fluorescent sensor for Cu^2+^ and Hg^2+^ detection with detection limits of 187.99 and 82.14 nM, respectively, was developed. Furthermore, the Au-loaded alginate-based fibers outperformed the pristine Ca-ALG fibers in terms of mechanical properties. Among the eleven common metal cations tested, Hg^2+^ cations entirely extinguished the fluorescence, whereas Cu^2+^ cations caused a noticeable drop in fluorescence intensity (Figure 4).

Another research studied alginate-based materials for water purification. Because cellulose/alginate’s molecular chain contains a lot of hydroxy and carboxylic acid groups, it has the potential to purify water by adsorbing metal ions and organic dyes through hydrogen and chelate bonding. A carbonized carboxymethyl chitosan/SA hydrogel evaporator was able to remove phenol with 95.37% efficiency and simultaneously desalinate water and remove volatile organic compounds using photocatalysis. The evaporation rate was 2.24 kg·m^−2^·h^−1^ [113].

Volatile aldehydes have a negative influence on both human health and the environment. Thus, a quick, simple, and highly accurate method for the simultaneous detection and removal of several aldehydes is widely awaited. Coating fluorescent alginate-modified surfactants (APGF and APOF) on ZIF-8 metal-organic frameworks (MOFs) resulted in the development of novel APGF@ZIF-8 and APOF@ZIF-8 sensing materials, which are porous fluorescent sensors (SBET up to 1519 m^2^/g). The developed sensors’ ability to detect acetaldehyde, formaldehyde, glyoxal, and benzaldehyde was assessed. With the limits of detection (LOD) values ranging from 0.001 to 0.18 μM (0.106–10.44 ppb), all aldehyde fluorescence spectra showed impressive linear relationships in the range of 0.05–200 μM. With good recovery rates of 96–77%, these sensors were effectively used to identify a variety of volatile aldehydes in river water samples. It is interesting to note that fluorescent APGF@ZIF-8/CS and APOF@ZIF-8/CS films were developed as disposable, portable methods to remove formaldehyde, acetaldehyde, benzaldehyde, and glyoxal from water. An exceptional formaldehyde adsorption capacity of 58.30 mg/g and an adsorption removal effectiveness of 93.5% was demonstrated for APOF@ZIF-8/CS [15].

**Hydrogen peroxide sensing:** Hydrogen peroxide (H_2_O_2_) sensing is a crucial aspect of various fields, including biomedical research and environmental monitoring. Accurate detection and measurement of H_2_O_2_ levels is vital for understanding its role in biological processes, diagnosing diseases, and monitoring environmental pollutants [114]. Alginate-based nanoparticles have depicted great potential in hydrogen peroxide sensing.

**Figure 5:**
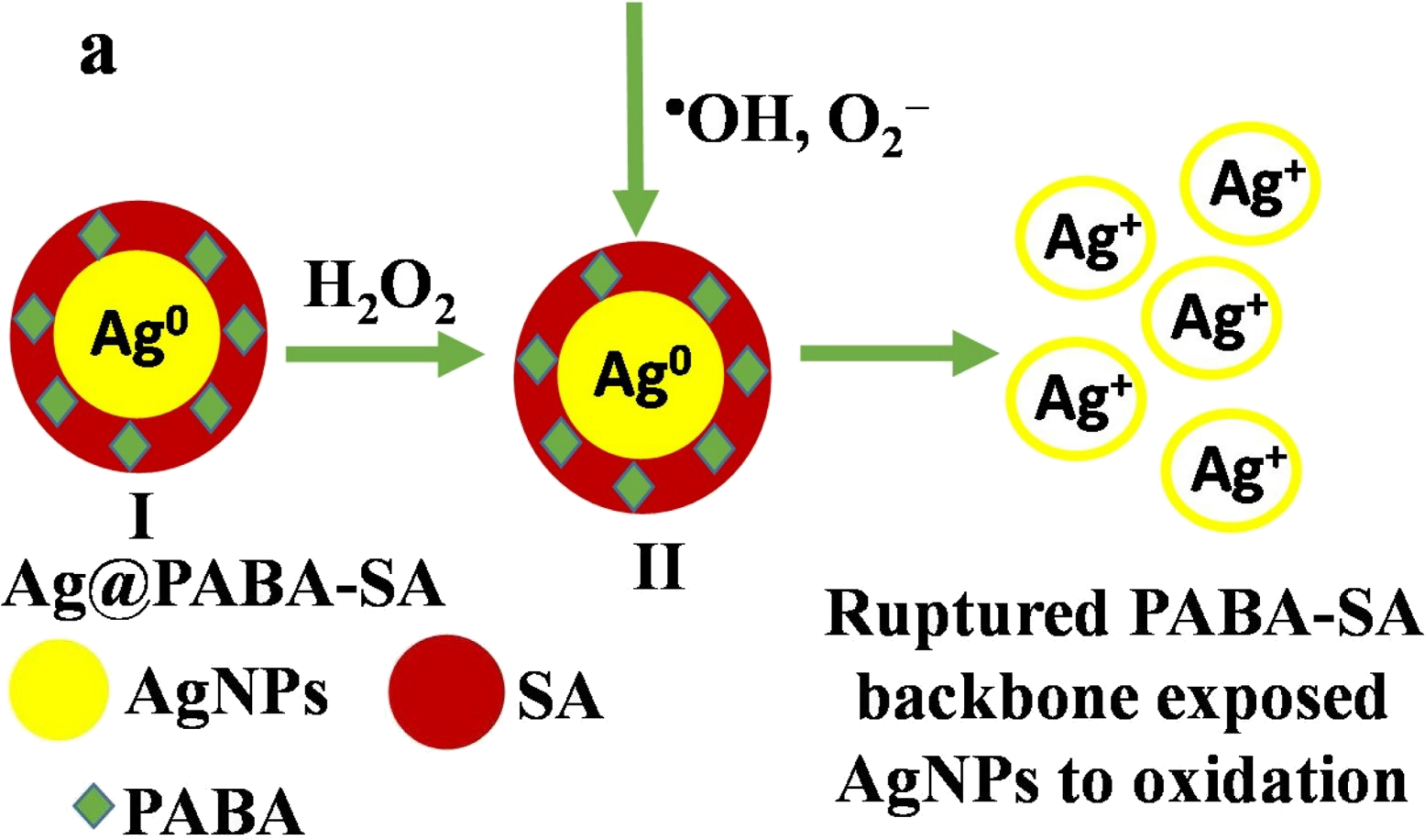
The mechanism of Ag@PABA-SA nanogel for detection of H_2_O_2_. Figure 5 was reprinted from [115], Carbohydrate Polymers, Vol. 246, by T. J. Jayeoye, T. Rujiralai, “Green, in situ fabrication of silver/poly(3-aminophenyl boronic acid)/sodium alginate nanogel and hydrogen peroxide sensing capacity”, 116657, Copyright (2020), with permission from Elsevier. This content is not subject to CC BY 4.0.

A silver/poly(3-aminophenyl boronic acid)/sodium alginate nanogel (Ag@PABA-SA) was synthesized using a green, in situ chemical oxidative polymerization approach for detecting hydrogen peroxide in lake water [115]. When this experiment was carried out without alginate, it showed unsuccessful results. Importantly, with alginate present, PABA covalently attached to the hydroxy groups of alginates to form a PABA-SA semi-interpenetrating network on which AgNPs were concurrently deposited (Figure 5). As a result, very stable polymer-based AgNPs were formed. The Ag@PABA-SA nanogel was tested as a colorimetric probe for detecting H_2_O_2_. The researchers obtained broad linearity between 5 and 1000 M H_2_O_2_, a low limit of detection (LOD) of 1.0 M, good accuracy (3.5%), recovery (95–105%), and high selectivity.

Gold nanoparticles loaded on a hydrogel based on alginate (Alg) and poly(3,4-ethylenedioxythiophene) showed excellent results in sensing hydrogen peroxide even at very small concentrations [116]. The synergistic combination of each component’s characteristics produced a novel flexible nanocomposite with an excellent ability to detect hydrogen peroxide, which was then used to detect ʟ-lactate oxidation. The hydrogel was used to detect hydrogen peroxide using cyclic voltammetry and chronoamperometry, with a linear response and detection limits of 0.91 M and 0.02 M, respectively. In most sensing applications, sodium alginate is used as a capping agent, which binds molecules to show its compatibility and antimicrobial properties. Oxidative and sensitive materials are loaded into sodium alginate gel, and the absence and concentration of hydrogen peroxide in water and milk can be analyzed by a colorimetric method [117].

**Figure 6:**
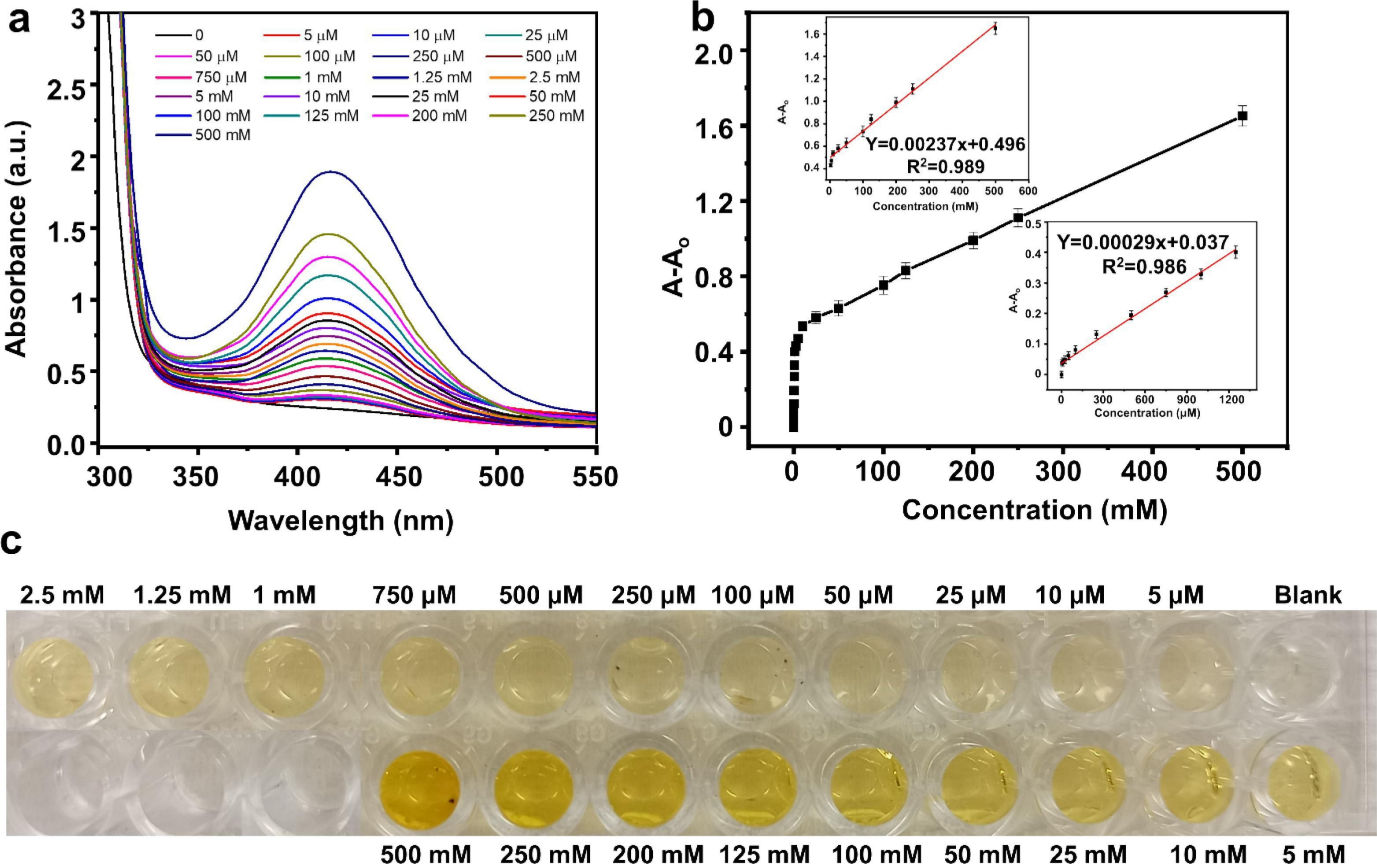
(a) UV–vis spectra for H_2_O_2_ detection by colorimetric solid hydrogel using Fe_3_O_4_@AMALG12@Ag nanocomposite. (b) Standard plot showing absorbance change as a function of H_2_O_2_ concentration (from 0 to 500 mM). The insets highlighted the linear detection range charts. (c) Colorimetric solid hydrogel for H_2_O_2_ with increasing H_2_O_2_ concentrations. Figure 6 was reprinted from [118], Chemical Engineering Journal, Vol. 453, by S. M. Ismail, A. A. Abd-Elaal, F. H. Abd El-salam, F. A. Taher, I. Aiad, S. M. Shaban, “Synthesis of silver decorated magnetic Fe_3_O_4_/alginate polymeric surfactant with controllable catalytic activity toward p-NP removal and enzymatic-mimic activity for solid-colorimetric H_2_O_2_ detection”, 139593, Copyright (2023), with permission from Elsevier. This content is not subject to CC BY 4.0.

In another work, an alginate hybrid silver magnetite nanocomposite (Fe_3_O_4_@AMALG12@Ag) with enhanced catalytic and enzymatic peroxidase mimicking activity was synthesized via self-assembly. The decorated Fe_3_O_4_@AMALG12@Ag was incorporated into an agarose hydrogel structure, and *o*-phenylenediamine (OPD) was used as a peroxidase substrate to detect H_2_O_2_ calorimetrically (Figure 6). The solid kit demonstrated a linear dynamic range of 0 to 1250 μM, with a LOD of 14 μM. Based on the selective enzymatic release of H_2_O_2_, the developed solid kit could be used to detect many biomarkers, including glucose, lactate, uric acid, and cholesterol [118].

**Humidity sensing:** One area that has shown promise in the field of humidity sensing is the use of nanoparticles, particularly alginate-based nanoparticles. One research presented an autoregulatory system for humidity sensing employing a nanoscale wrinkle-patterned hydrogel with programmable feedback cycle. The wrinkled pattern monitors humidity fluctuations, and a plasmonic nanoparticle lattice regulates wrinkling by plasmonic heating [119]. Another study compared the humidity sensing capabilities of ZnO nanoparticles and nanotetrapods and found that the nanotetrapods were five times more sensitive to humidity than the nanoparticles [120].

The humidity sensing capabilities of alginate-based nanoparticles have been explored in various studies. One study by Han et al. [121] suggested to use a low-cost and environmentally friendly surface acoustic wave (SAW) humidity sensor made of sodium alginate (SA) hydrogel. The hydroxy and carboxylate groups can easily adsorb H_2_O molecules to generate hydrogen bonds. These adsorbed H_2_O molecules considerably improve the SAW sensor’s mass loading and signal responses. The authors compared SEM images and frequency responses, and they found that a single layer of sodium alginate film is best for this SAW humidity sensor.

Another fluorescence-based smart sensor from alginate nanofilms was prepared [122]. Scientists proved that relative humidity could be detected using a guar gum–sodium alginate (GGSA) nanocomposite film. The study found that the fluorescence of biocomposite films under UV light varies at various relative humidity levels. Fluorescence quenching occurs at high relative humidity, whereas the fluorescence intensity in the nanocomposite film rises at low humidity. A fluorescence spectrophotometer was used to examine the change in fluorescence intensity of the nanocomposite film at different relative humidity levels.

**Figure 7:**
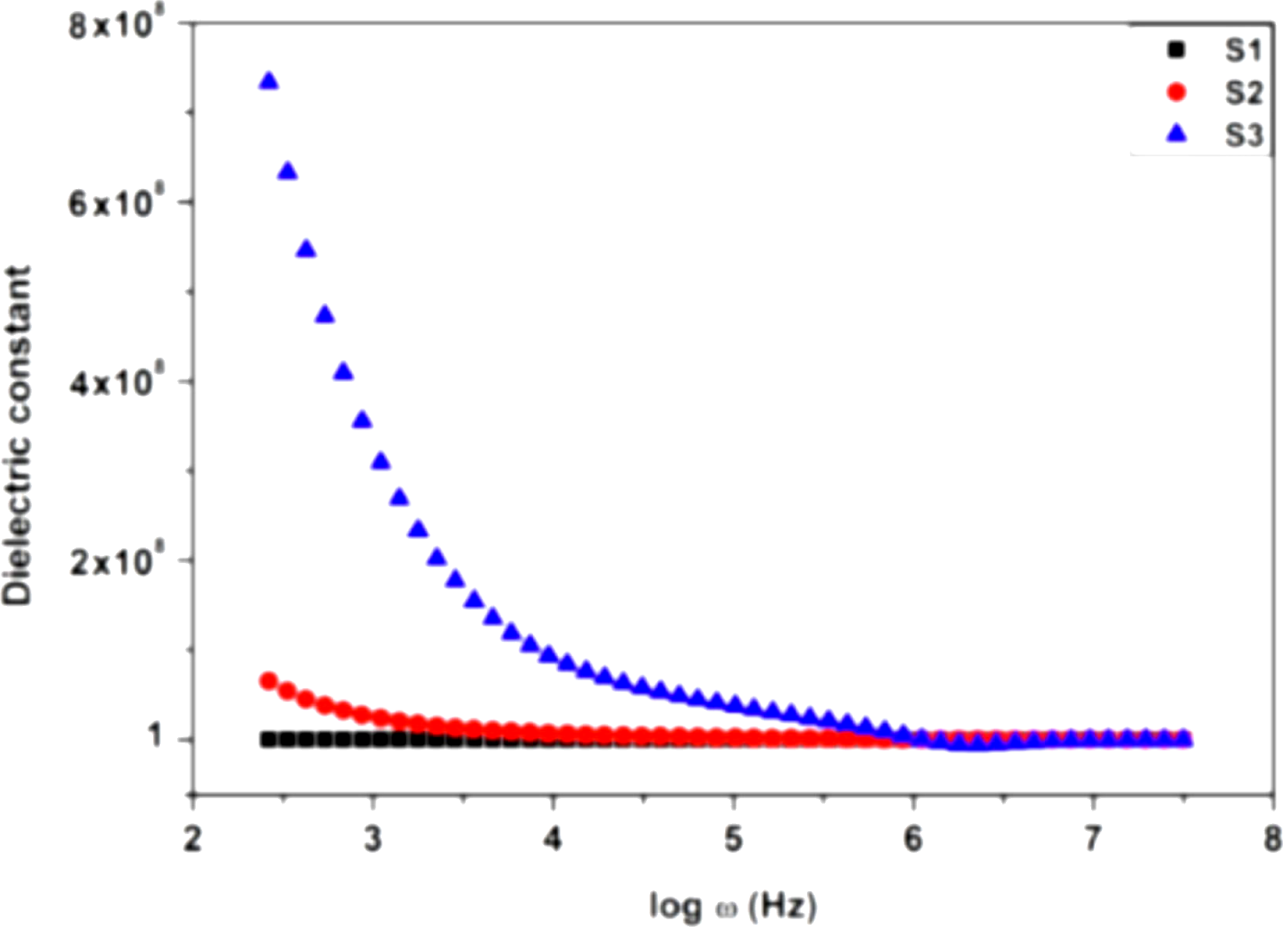
Dielectric measurement results of the combined polymer. S_1_ – clean film, S_2_ – low moisture content biopolymeric film, S_3_ – high moisture content biopolymeric film. Figure 7 was reprinted from [123], Materials Today: Proceedings, Vol. 64, by T. Thiruganasambanthan, S. M. K. Thiagamani, C. Santulli, S. Krishnasamy, C. Muthukumar, “Preparation of Sodium Alginate/Rice starch blend polymer film for soil moisture sensing”, pages 352–356, Copyright (2022), with permission from Elsevier. This content is not subject to CC BY 4.0.

Alginate can be applied to moisture sensing in soil, which is good for the agricultural sector. The moisture-sensing ability of alginate-based biopolymers was demonstrated by Indian researchers [123]. A nanocomposite from sodium alginate and starch was prepared by solution casting [124]. The polymer film’s soil moisture sensing capabilities were proved by measuring the dielectric constant of the polymer with soil samples containing low and high moisture (Figure 7). According to the results, the polymer film has excellent moisture sensing characteristics at frequencies as low as a few kilohertz.

Another alginate-based humidity sensor is carbonized lignin-loaded sodium alginate (CL/SA). The CL/SA composite film provided extremely high sensitivity, low hysteresis, and consistent repeatability throughout a wide relative humidity range of 11–97%. Furthermore, the CL/SA sensor allowed for measuring human breathing. According to the results, CL/SA composite is a good humidity-sensitive material [125].

However, further research is needed to fully understand and optimize the properties of alginate-based nanoparticles for environmental sensing. Future studies should focus on optimizing the stability, porosity, and degradation rate of alginate-based nanoparticles in order to enhance their sensing capabilities. Additionally, efforts should be made to explore the potential of alginate-based nanoparticles for detecting a wide range of environmental pollutants, such as heavy metals and pesticides.

### Biosensors

#### Biomedical applications of alginate nanoparticles

Biosensors identify biological materials or alterations in biological activity. Alginate-encapsulated enzymes have attracted a lot of attention lately and are a potential advancement in the field of biosensors. Alginate-encapsulated enzymes provide several benefits, including excellent stability and extended storage life, which makes them a desirable choice for biosensor applications. Additionally, they may be quickly immobilized on a surface, such as a glass electrode, which increases their sensitivity and precision for identifying target analytes [126].

The biosensing capabilities of alginate-based nanoparticles have been established by several studies. For example, glucose oxidase encapsulated in an alginate biopolymer can detect glucose levels in human blood samples with excellent precision and sensitivity. Similarly, scientists created an alginate–CuO–glucose oxidase-based biosensor for glucose detection, which demonstrated high repeatability and long-term stability. Another research used horseradish peroxidase coated in calcium alginate beads to detect hydrogen peroxide in ambient water samples [127]. Ionotropic alginate hydrogel biosensors generally function by allowing the analyte to diffuse into the hydrogel’s free volume, where biomolecular binding is turned into a measurable signal [128]. Overall, alginate-based biosensors offer enormous promise for biological applications because of their simplicity, low cost, and excellent sensitivity and selectivity. The table below depicts biomedical applications of alginate-based nanomaterials (Table 2).

**Table 2:** Selected studies on Alginate-based nanoparticles in sensing techniques for biomedical applications.

Biological molecule	Transducer	Method	Linear range	LOD	Applications	Ref.

Escherichia coli bacteria	alginate-methacrylate hydrogel	green fluorescent protein (GFP) expression	1 × 10^−5^–1 × 10^−7^ M	–	quorum sensing (pseudomonas aeruginosa)	[129]
glucose	copper nanoclusters (CuNCs) loaded alginate gel network with Ca^2+^	photoluminescence intensity	0.1–2.0 mM	3.2 × 10^−5^ M	sensor for glucose	[130]
glucose	TiO_2_ loaded alginate hydrogel	rapid colorimetric detection	0.1–1 mM for lactate, 0.1–0.8 mM for glucose	0.069 mM for lactate, 0.044 mM for glucose	detection of sweet biomarkers	[131]
silica-glucose oxidase nanoparticles (SiO_2_-GOx)	Fe^3+^-alginate hydrogel	Fenton reaction	1 pg·mL^−1^ to 100 ng·mL^−1^	0.447 pg·mL^−1^	“smart” electrochemical sensing. Neuron-specific enolase (NSE) quantification	[132]
ascorbic acid	CuO trapped alginate	amperometric measurement	10–150 μM	1.97 μM	detection of ascorbic acid in sweat	[133]
organophosphorus pesticides	acetylcholinesterase (AChE)-mediated alginate hydrogel	diameter measurement of spot on the filter paper	up to 66.7 ng/mL	3.3 ng/mL	quantitative determination of organophosphorus pesticides	–
penicillinase	bromothymol blue-cetyltrimethylammonium bromide-alginate complex	dual-readout paper-based sensor	8.00 × 10^−3^–2.67 mU/μL for distance-readout	2.67 × 10^−3^ mU/μL for distance-readout	smartphone-based detection of penicillinase	[134]
bacillus anthracis	europium ions were employed to Quantum dots and alginate	green fluorescent film	1–45 µM	0.17 µM	dipicolinic acid (DPA) detection	[135]
glucose oxidase (GO)	sodium alginate	diameter measurement of spot on the filter paper	between 1.4–7.0 mM	1.4 mM	determine glucose in fruit samples	[136]
serum-HER2 and serum-CA125	porous calcium alginate bead	multiplex detection	–	0.004 ng·mL^−1^ for serum HER2, 0.005 U·mL^−1^ for serum CA125	early detection of breast cancer	[137]

**Glucose detection via alginate nanomaterials:** Nanoparticles are an attractive choice for developing highly efficient biosensors [138–139]. Alginate-based nanoparticles can be modified to meet the specific needs of glucose detection, such as incorporating glucose oxidase for enzymatic sensing, allowing for efficient and accurate glucose detection [140]. The fabrication of alginate-based nanoparticles for glucose detection involves several steps. First, alginate is mixed with a cross-linking agent to form a gel-like solution. Then, glucose oxidase is incorporated into the alginate mixture. Next, the mixture is sonicated to evenly distribute the alginate-based nanoparticles in an aqueous solution. Once the nanoparticles are formed, they can be immobilized onto a substrate or electrode for glucose detection [131]. The high colloidal stability of alginate-based nanoparticles ensures their long-term stability and prevents aggregation, leading to more reliable and consistent glucose detection results [141]. Alginate-based nanoparticles can be easily functionalized to enhance their performance in glucose detection. For instance, the surface of alginate-based nanoparticles can be modified with specific receptors or ligands that selectively bind to glucose molecules, improving the sensitivity and selectivity of the biosensor. Moreover, alginate-based nanoparticles have the potential for controlled release of insulin in response to glucose levels. This capability makes them particularly promising for the development of glucose-responsive systems for insulin delivery, which could revolutionize diabetes management [142]. Glucose biosensing can be performed by two methods, namely, enzymatic and non-enzymatic electrochemical biosensing. In enzymatic electrochemical sensors, oxidative enzymes can be immobilized in the alginate matrix [78]. Non-enzymatic sensors for glucose rely on the direct electrochemical oxidation of glucose. Materials for both enzymatic and non-enzymatic glucose biosensors include conductive polymers, enzymes, carbon nanotubes, and molecularly imprinted polymers (MIPs). MIPs function similarly to enzymes, producing polymeric cross-linked active sites for certain analytes. MIPs were initially used in optical sensing, but they have lately been examined in electrochemical glucose sensing [143].

**Figure 8:**
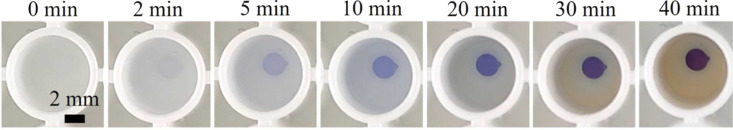
Photographs of the alginate/TiO_2_ scaffold for the detection of glucose (10–1000 µM) at different time ranges. Figure 8 was adapted from [144] (© 2023 S. Garcia-Rey et al., published by Elsevier, distributed under the terms of the Creative Commons Attribution-Non Commercial-No Derivs 4.0 International License, https://creativecommons.org/licenses/by-nc-nd/4.0/). This content is not subject to CC BY 4.0.

Sodium alginate can be used for both enzymatic and non-enzymatic electrochemical sensors. One study showed great potential of TiO_2_-loaded alginate nanoparticles in sensing of glucose in artificial sweat [144]. Scientists achieved fast detection time of glucose by encapsulation of TiO_2_ nanotubes inside the alginate sensing scaffold. In this experiment, the physiological range of glucose from 10 to 1000 µM was tested using colorimetry (Figure 8). A chrono-sampling microfluidic device was used to detect the change of glucose levels over time. This new device based on alginate nanoparticles might be further developed as a tracking device for sweat glucose detection.

**Figure 9:**
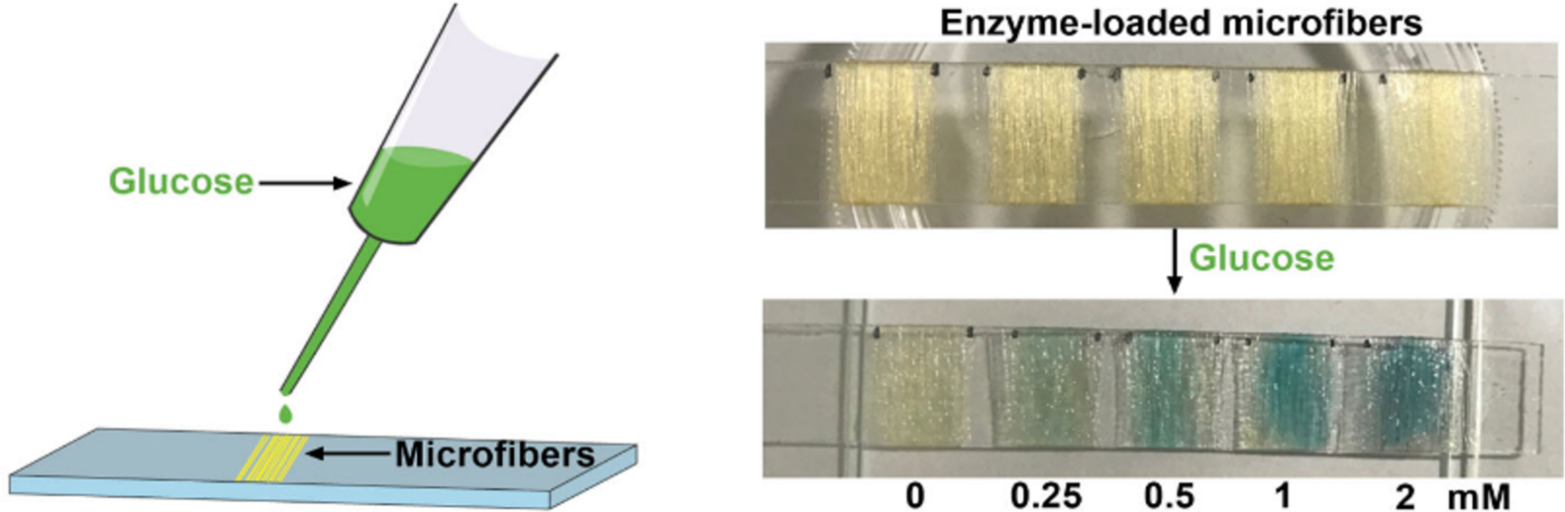
Detection of different concentrations of glucose (0, 0.25, 0.5, 1, 2 mM) with enzymes and TMB-loaded microfibers. Figure 9 was adapted from [145], W. Zhang et al., “Microfluidic fabrication of tunable alginate-based microfibers for the stable immobilization of enzymes”, Biotechnology Journal, with permission from John Wiley and Sons. Copyright © 2022 Wiley-VCH GmbH. This content is not subject to CC BY 4.0.

**Figure 10:**
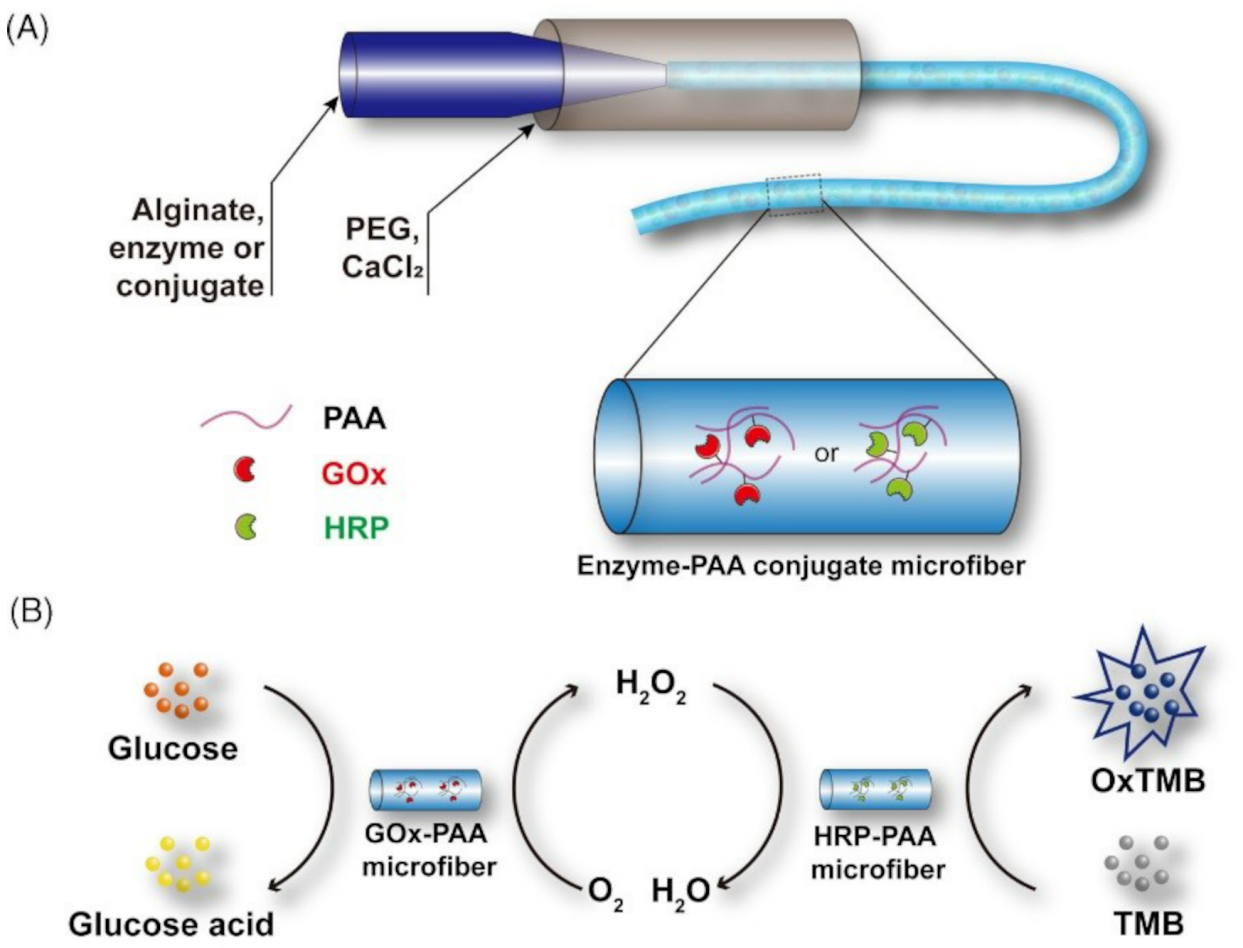
(A) Diagram for the enzyme-immobilized alginate microfibers and (B) Microfiber-based colorimetric detection for glucose concentration. Figure 10 was adapted from [145], W. Zhang et al., “Microfluidic fabrication of tunable alginate-based microfibers for the stable immobilization of enzymes”, Biotechnology Journal, with permission from John Wiley and Sons. Copyright © 2022 Wiley-VCH GmbH. This content is not subject to CC BY 4.0.

Another microfluidic fiber-based enzymatic biosensor was developed. Microfibers encapsulated bovine serum albumin (BSA), glucose oxidase (GOx), and horseradish peroxidase (HRP) efficiently (Figure 9). In contrast, physically adsorbed enzymes are quickly disseminated throughout the catalytic reaction system. Enzyme leakage may be greatly decreased by incorporating enzymes into alginate-based microfibers, resulting in stable immobilization, higher recyclability, and improved thermostability. Furthermore, using the cascade reaction of these enzymes, GOx and HRP-loaded microfibers were synthesized under optimum circumstances for the visual detection of glucose, displaying a color change in response to glucose in a concentration range of 0–2 mM (Figure 10) [145].

**Figure 11:**
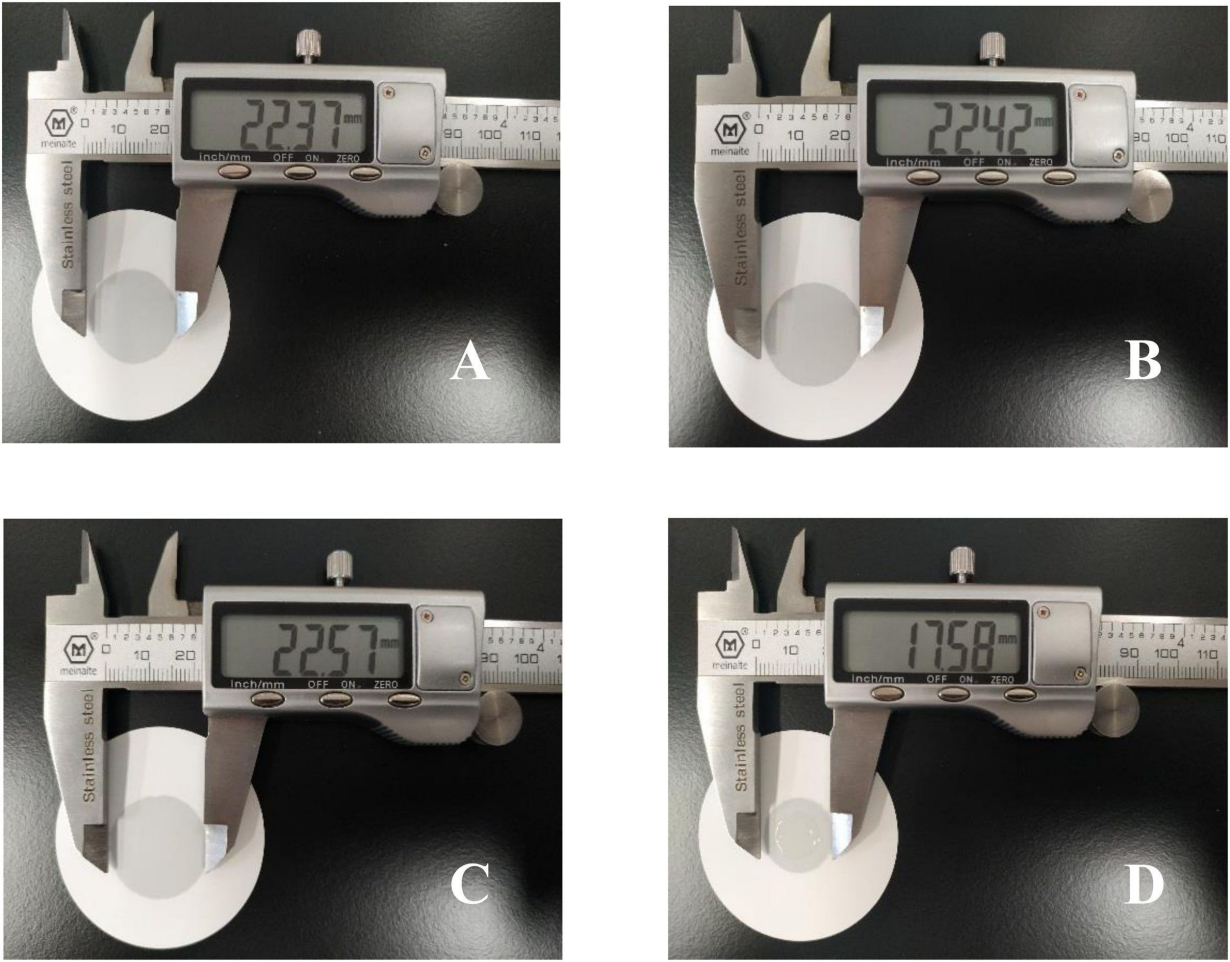
Diameter measurement of different solutions on the membrane. A: Alginate solution; B: Alginate solution with CaCO_3_; C: Alginate solution with CaCO_3_ and glucose oxidase; D: Alginate solution with CaCO_3_, glucose oxidase and glucose. Figure 11 was reprinted from [136], Enzyme and Microbial Technology, Vol. 148, by H. Zhang, X. Li, Z.M. Qian, S. Wang, F. Q. Yang, “Glucose oxidase-mediated sodium alginate gelation: Equipment-Free detection of glucose in fruit samples”, 109805, Copyright (2021), with permission from Elsevier. This content is not subject to CC BY 4.0.

Non-enzymatic electrochemical sensing is more cost-effective and has a longer lifespan than enzymatic sensing [146]. Because of the difficult enzyme immobilization processes, poor chemical and thermal durability, expensive manufacturing, and other limitations of enzymatic sensors, researchers have begun to investigate the feasibility of non-enzymatic biosensors for glucose sensing. Stability is a critical challenge for enzymatic sensors since enzymes can get denatured because of immobilization, decreasing their function and shelf-life. Furthermore, enzymes are easily impacted by several environmental changes including humidity, temperature, pH, and hazardous substances. As a result, additional research is being conducted on non-enzymatic electrochemical sensors because of various advantages such as cost, stability, simple fabrication processes, and reproducibility [147]. Glucose sensing can be performed by non-enzymatic sensors, and the detection of glucose can be measured by electrocatalytic oxidation [148]. Alginate-based composite gels were investigated for glucose sensing, demonstrating their utility as excellent electrode materials for sensors [136]. Zhang et al. [136] developed a glucose assay kit based on sodium alginate. The scientists made use of the fact that the gelation of alginate is highly pH-dependent. When glucose is added to a CaCO_3_-and GOx-containing alginate solution, GOx is oxidized, releasing gluconic acid and H_2_O_2_. The pH of the solution decreases above a certain acid concentration to a level suitable for alginate cross-linking, increasing the viscosity of the hydrogel. Monitoring the diameter of microporous membranes allowed the authors to determine the viscosity of the hydrogel, and consequently the glucose content in the test samples, within the range of 1.4–7.0 mM (Figure 11). The suggested paper-based sensor is a simple, low-cost, and accurate technique that provides a reference test toward the on-site glucose detection in fruit samples without complex apparatus [136].

Chaudhari et al. discussed the use of alginate nano-microspheres loaded with glucose oxidase to create fluorescence-mediated glucose detection biosensors. The operation is based on fluorescence quenching of near-infrared radiation (NIR) of the oxygen-sensitive dye [149].

**Figure 12:**
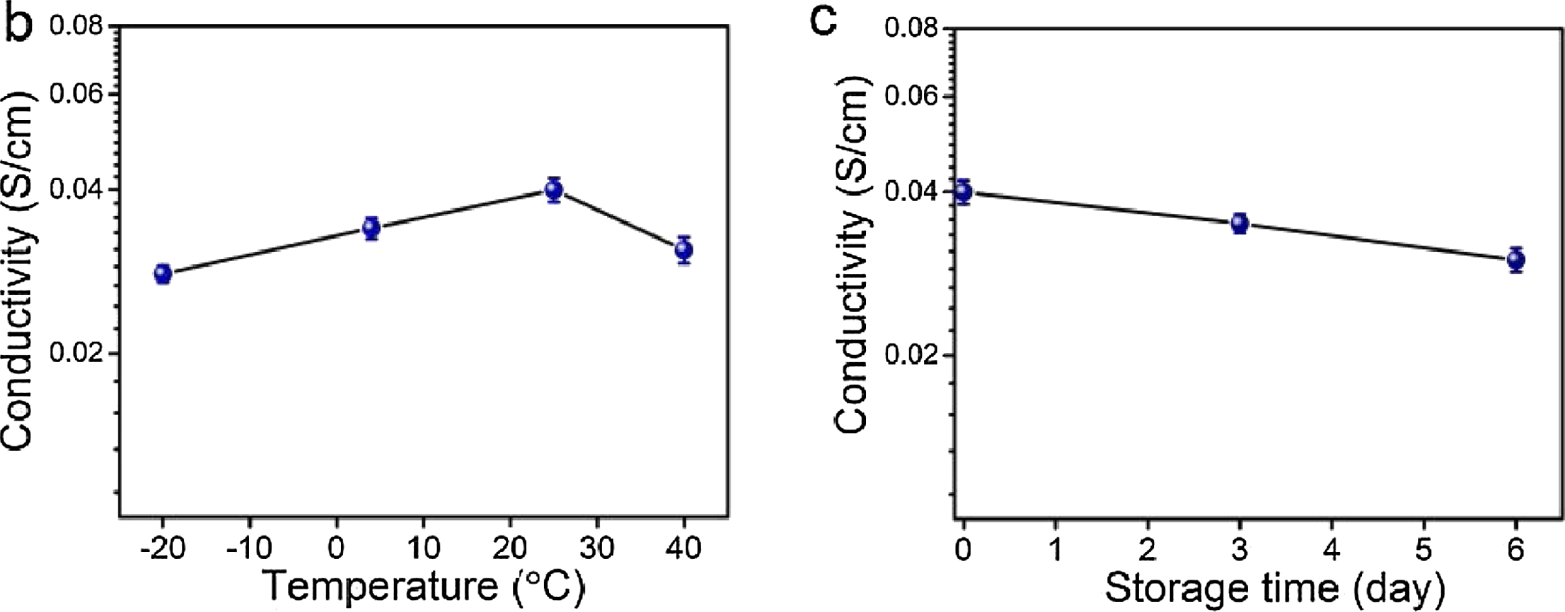
Dependence of conductivity on temperature (b); Dependence of conductivity on storage time (c). Figure 12 was reprinted from [150], Carbohydrate polymers, Vol. 235, by H. Chen, Y. Gao, X. Ren, G. Gao, “Alginate fiber toughened gels similar to skin intelligence as ionic sensors”, 116018, Copyright (2020), with permission from Elsevier. This content is not subject to CC BY 4.0.

**Human skin performance sensing by alginate-based nanoparticles:** Chen et al. [150] created alginate fiber gels for a skin-like ionic sensor. The sodium alginate fibers induced by incorporating NaCl into polyvinyl alcohol (PVA) and glycerol were used to create gels with improved mechanical performance. This conductive gel demonstrated sensory qualities towards stress and strain, allowing it to detect electric signals under various pressures. Simultaneously, the gels were created as sensors to detect various human body movements (including finger, arm, knee, sole, and throat) and even recognized diverse gestures and sign language. Gels demonstrated temperature resistance ranging from −20 to 40 °C and non-drying qualities at 25 °C for 6 days. To respond to the complex and changing external conditions, the gels displayed attractive remoldability. The results showed that the gels performed similarly to skin in all areas. Soft robots, artificial skins, and smart wearable devices are possible uses (Figure 12).

**Cancer sensing applications of alginate-based nanoparticles:** Cancer remains one of the leading causes of morbidity and mortality worldwide. It poses a substantial burden to public health and the economy, particularly with the growing aging population. In recent years, there has been a growing interest in the development of nanoparticles for cancer precision medicine. Among the various biomedical strategies, nanoparticles have garnered significant attention in cancer therapy to deliver drugs specifically to cancer cells [151]. Alginate nanoparticles can be modified and functionalized to enhance their cancer-sensing capabilities [152]. Zhang et al. [153] reported the development of functionalized alginate nanoparticles that can deliver antigens to dendritic cells for cancer immunotherapy. These nanoparticles were coated with antibodies specific to dendritic cell surface markers, enabling targeted delivery and activation of the immune system against cancer cells. The main role of alginate nanoparticles for cancer sensing is to encapsulate and deliver fluorescent and other biomarkers to cancer cells [154]. These nanoparticles can be administered systemically and accumulate specifically in tumor tissues, allowing for non-invasive imaging and early detection of cancer. Moreover, alginate-based nanoparticles have been used to create multifunctional platforms for cancer therapy. For example, alginate nanogels loaded with gold nanoparticles have been developed as a thermoresponsive platform for chemo-photothermal therapy for breast cancer [155].

One study reported that the specificity and sensitivity of alpha-fetoprotein (AFP) can be improved by alginate nanomaterials [156]. AFP is the most widely used biomarker for the early diagnosis of hepatocellular carcinoma (HCC). The detection of AFP is determined by the change in fluorescence intensity at 349 nm of AFP. AFP was placed in alginate, and the sensor proved its appropriateness for AFP measurements with 93.33% sensitivity and 90.0% specificity.

In another study, scientists carried out research on alginate beads for early diagnosis of breast cancer [137]. Because various cancer biomarkers may be expressed at different stages of the disease, early cancer detection requires a sensitive biosensor capable of simultaneously detecting tiny amounts of numerous cancer biomarkers in clinical samples (multiplex analysis). The calcium alginate immuno-beads’ large surface area-to-volume ratio improved the sensitivity and linear dynamic range of the proposed multiplex analysis approach significantly. With a LOD of 0.004 ng·mL^−1^ for serum HER2, which is below clinical cutoff values, this sensing application allows for early diagnosis of breast cancer. Furthermore, this analysis takes 30 min and the resulting beads have good stability (at least 14 days).

Alginate-based hydrogel can be used for 3D printing of cancer marker genes. Scientists studied a 3D-printed scaffold for the determination of breast cancer reporter cells. Alginate was employed as a basis material to build scaffolds consisting of a 3D grid with periostin and hydroxyapatite. During the research, different concentrations of alginate (8–15%) were tested. It was found that there is no difference between lower and higher concentrations. Results showed that alginate is a suitable material for the experiment and can be a relevant model for drug discovery and diagnosis of breast cancer [157].

Cancer sensing can be performed by intercellular and extracellular pH changes. It has been demonstrated that normal cells have an intracellular pH of 7.2 and an external pH of 7.4, but cancer cells have a lower intracellular pH of 6.7–7.1 [158]. Therefore, scientists constructed highly pH-sensitive nanosensors based on alginate [22]. Alginate-based pH-responsive nanosensors can detect tiny pH changes in a variety of biological systems without inflicting major harm. This pH sensing technique uses natural polymers and pH-sensitive fluorophores in an organic, solvent-free, and environmentally friendly manner. Scientists used multiple chemical conjugation techniques to attach fluorophores to alginate polymer chains, resulting in pH nanosensors with an increased dynamic range, which can detect pH changes ranging from 3.5 to 7.5. They strongly suggested to use the alginate nanosensor for intracellular pH detection and other biological systems [22].

**Figure 13:**
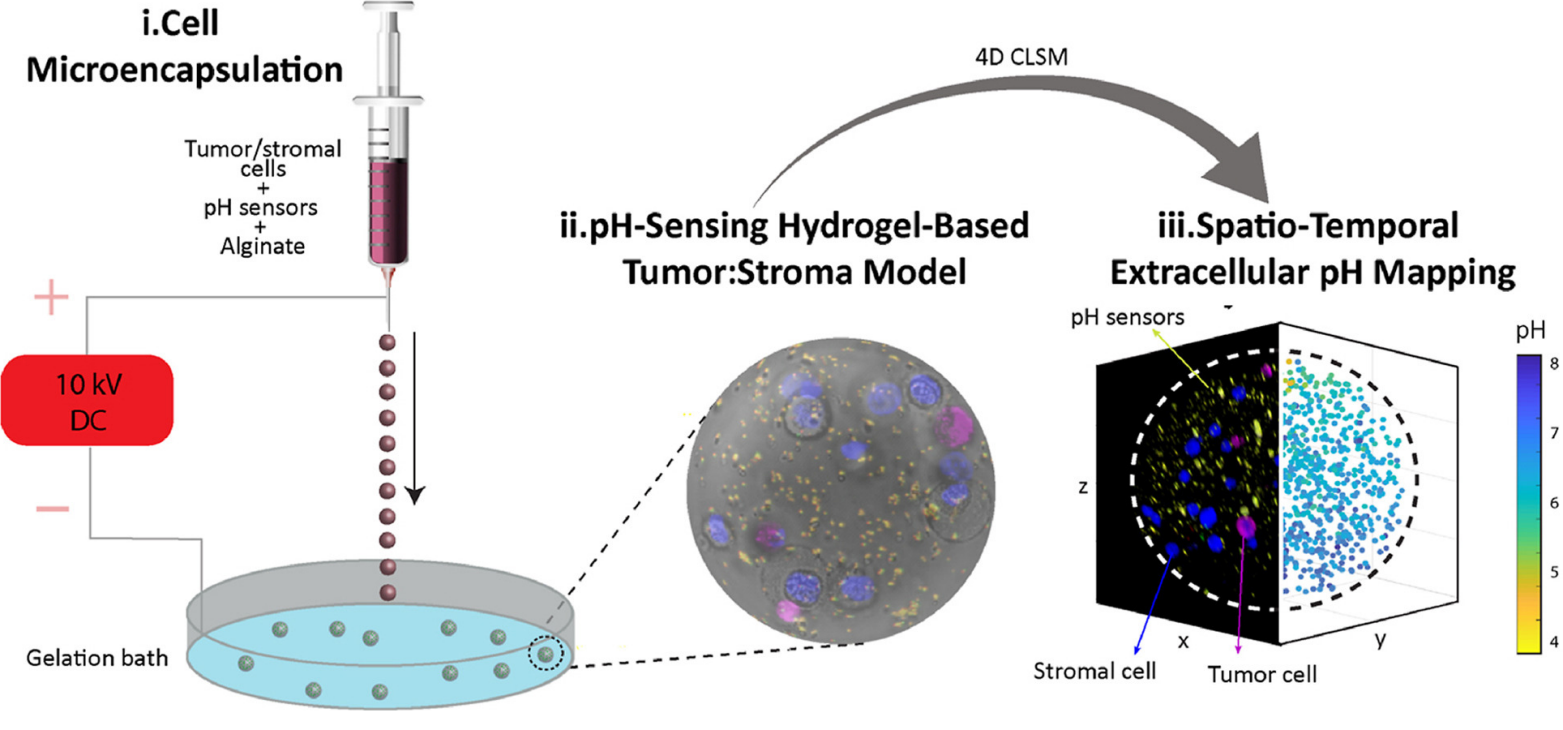
A system for sensing extracellular pH changes by fluorescent sensor-loaded alginate nanoparticles. Figure 13 was reproduced from [159] (© 2022 R. Rizzo et al., published by Elsevier, distributed under the terms of the Creative Commons Attribution 4.0 International License, https://creativecommons.org/licenses/by/4.0).

In another research, a pH-sensing 3D scaffold was developed based on an alginate matrix for determining extracellular pH changes [159]. The authors encapsulated silica inside alginate-based 3D microgels in order to make fluorescent pH sensors. Time-lapse imaging of 3D alginate microgels was carried out on live cells by confocal laser scanning microscopy, and the extracellular pH metabolic fluctuations were observed in both in vitro 3D mono- and 3D co-cultures of cancer and stromal pancreas cells. The authors measured the extracellular pH and concluded that under carefully regulated experimental circumstances, the device can detect pH changes in real time and numerous living cell types in a 3D environment. As a result, this technique may be used to identify cancer cells at early stages (Figure 13).

Overall, the use of alginate-based nanoparticles in cancer sensing holds great potential for improving diagnosis, treatment, and monitoring of cancer. These nanoparticles offer a versatile and efficient system for non-invasive imaging. Furthermore, their biocompatibility, biodegradability, low cost, and ease of production make them an ideal material for the development of nanoparticles with a wide range of applications in cancer research and therapy.

#### Pharmaceutical applications of alginate

In recent years, alginate-based nanoparticles have emerged as promising candidates for pharmaceutical sensing applications. These nanoparticles can be functionalized with specific sensing elements, such as sensors, dyes, or enzymes, to detect and quantify specific molecules or analytes of interest in the pharmaceutical field. The following Table 3 describes drug sensing applications of alginate-based nanoparticles.

**Table 3:** Selected studies on Alginate-based nanoparticles in drug sensing for pharmaceutical applications.

Drug	Transducer	Method	Linear range	LOD	Applications	Ref.

tetracycline	carbon dots in alginate hydrogel	the fluorescent emission intensity	1–20 µM	2 µM	tetracycline detection	[160]
theophylline	sodium alginate/multiwalled carbon nanotubes	electrode modification	0.01–60.0 μM	3.2 nM	determination of theophylline	[161]
morphine	ʟ-lysine, activated carbon and alginate	electrode modification	0.1–1000.0 μM	48 nM	determination of morphine	[162]
thrombin	metalloporphyrinic metal-organic framework (Cu-TCPP(Co) MOFs) with alginate	amplification of chemiluminescent signal	8.934 × 10^−13^ to 5.956 × 10^−10^ mol/L	2.178 × 10^−13^ mol/L	detection of thrombin in body fluids	[163]
paracetamol	alginate-modified cassava fibers	electrode modification	10–370 μM	1.2 μM	detection of Paracetamol	[164]
doxorubicin	silver nanoparticles with alginate layer	cyclic voltammetry (CV)	0.1–5.0 μg/mL	15 ng/mL	quantification of DOX in patient samples	[165]
palladium (II)	Au nanoparticles loaded alginate	polymer film sensing	0.05−2.10 μg·mL^−1^	0.038 μg·mL^−1^	determination of palladium in dental amalgam	[166]
alcohol oxidase	calcium alginate	electrochemical transducer	0–1.25 μg·mL^−1^	4.5 nA	analyze the levels of ethanol in whole blood samples	[167]

One of the key advantages of alginate-based nanoparticles in sensing applications is their biocompatibility and low toxicity. This makes them suitable for in vivo sensing, where they can be employed for real-time monitoring of drug levels or biomarkers in the body. For example, alginate-based nanoparticles loaded with fluorescent dyes or enzymes can be used as biosensors for detecting specific disease biomarkers or therapeutic drug concentrations in the bloodstream [168].

**Figure 14:**
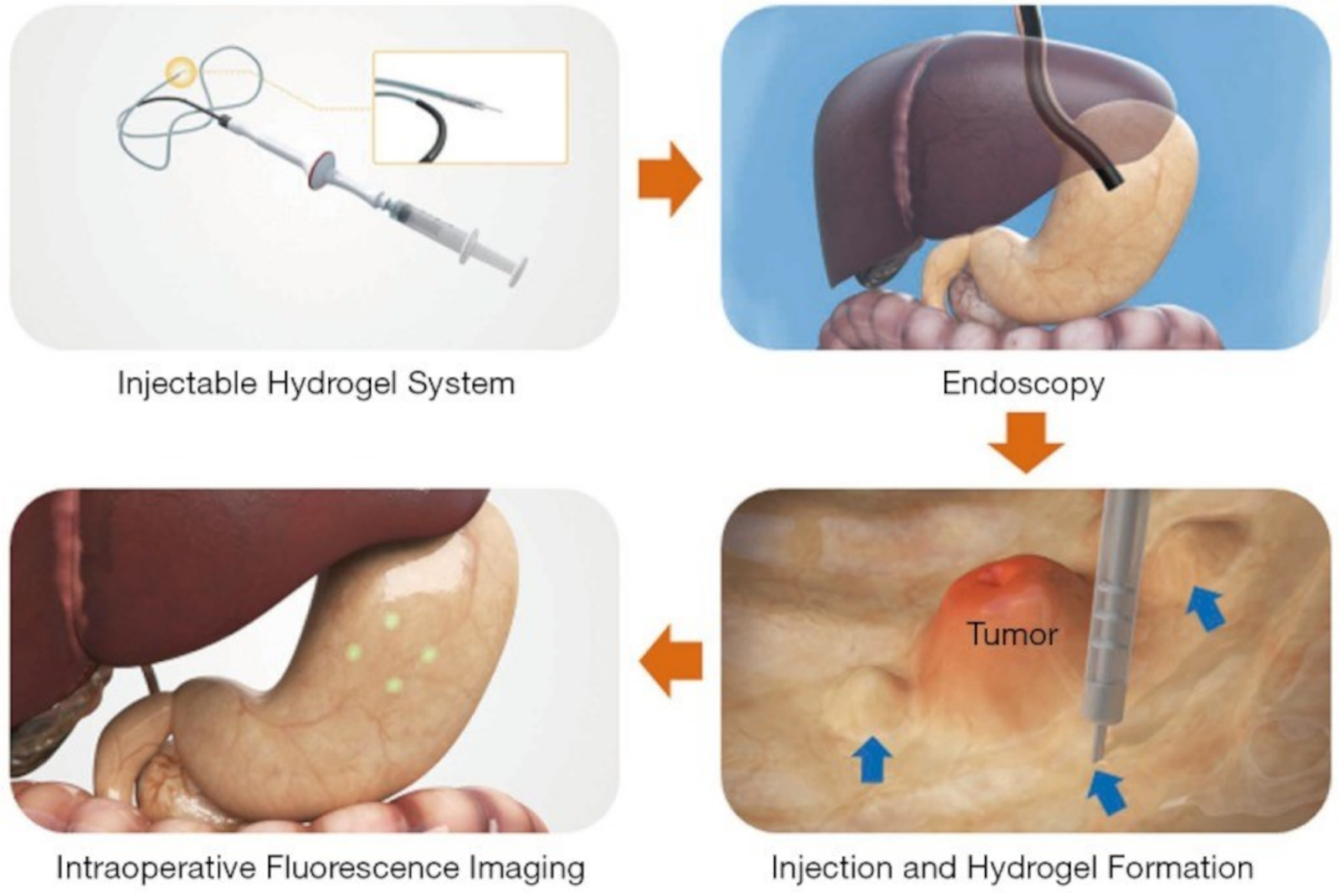
Usage of ICG-HSA-loaded alginate hydrogel for marking surgical sites and detection of the marked sites with fluorescence in real time. Figure 14 was reproduced from [169] (© Quantitative Imaging in Medicine and Surgery. All rights reserved, published by AME Publishing Company, distributed under the terms of the Creative Commons Attribution-Non-Commercial-No-Derivs 4.0 International License, https://creativecommons.org/licenses/by-nc-nd/4.0/). This content is not subject to CC BY 4.0.

Moreover, the porous nature of alginate nanoparticles yields high drug loading capacity, enhancing the sensing capability [67]. The nanoparticles can be engineered to encapsulate drugs or biomarkers within the alginate matrix, providing a controlled release of the analyte for detection. This controlled release can be triggered by specific stimuli, such as pH or temperature changes, resulting in a responsive and targeted sensing system. For example, alginate hydrogel can be applied to detect the location of cancer cells during surgery. Using alginate hydrogels filled with human serum albumin (HSA) and indocyanine green (ICG), a dye frequently used for diagnostic imaging, researchers created a device to enhance laparoscopic procedures. In a porcine model, the hydrogel’s efficacy as a fluorescent surgical marker was examined. The fluorescent marker was injected into the submucosal areas of the porcine stomach, and it remained there for three days without dispersing. Thus, it has been demonstrated that this laparoscopic fluorescence imaging technology (Figure 14) is a viable strategy for precisely detecting the site of cancer cells during surgery [169].

**Figure 15:**
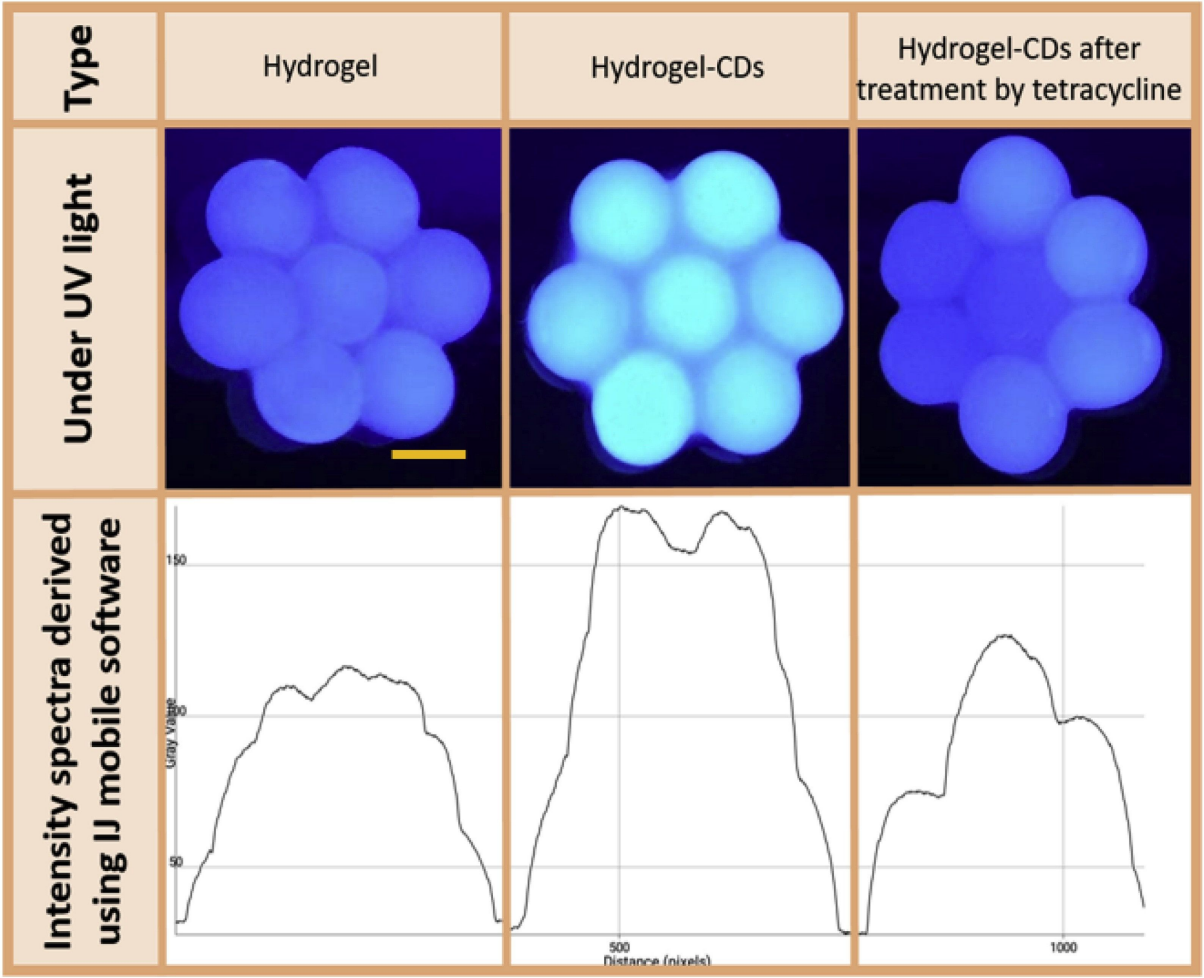
Images of hydrogel granules were obtained with the smartphone camera, and their spectrum was computed by a software program. Figure 15 was reprinted from [160], Journal of Environmental Chemical Engineering, Vol. 7, by H. Ehtesabi, S. Roshani, Z. Bagheri, M. Yaghoubi-Avini, “Carbon dots—Sodium alginate hydrogel: A novel tetracycline fluorescent sensor and adsorber”, 103419, Copyright (2019), with permission from Elsevier. This content is not subject to CC BY 4.0.

Another advantage of alginate-based nanoparticles is their ability to protect the encapsulated drugs or biomarkers from degradation [170], ensuring accurate and reliable sensing. Additionally, surface modifications of these nanoparticles with targeting ligands or antibodies allows for specific recognition and binding to disease-specific cells or molecular targets, improving the sensitivity and selectivity of the sensing system. One study suggested using an alginate-based sensor for the determination of tetracycline (TC). Researchers encapsulated carbon dots (CDs) as a fluorescent-based sensor for TC in alginate hydrogel [160]. Using a smartphone-based fluorimeter, the degree of fluorescence quenching of the carbon dots in the hydrogel structure was assessed in the presence of TC. The outcomes demonstrated that very little concentrations of TC might lowered the fluorescence intensity of CDs (Figure 15).

**Figure 16:**
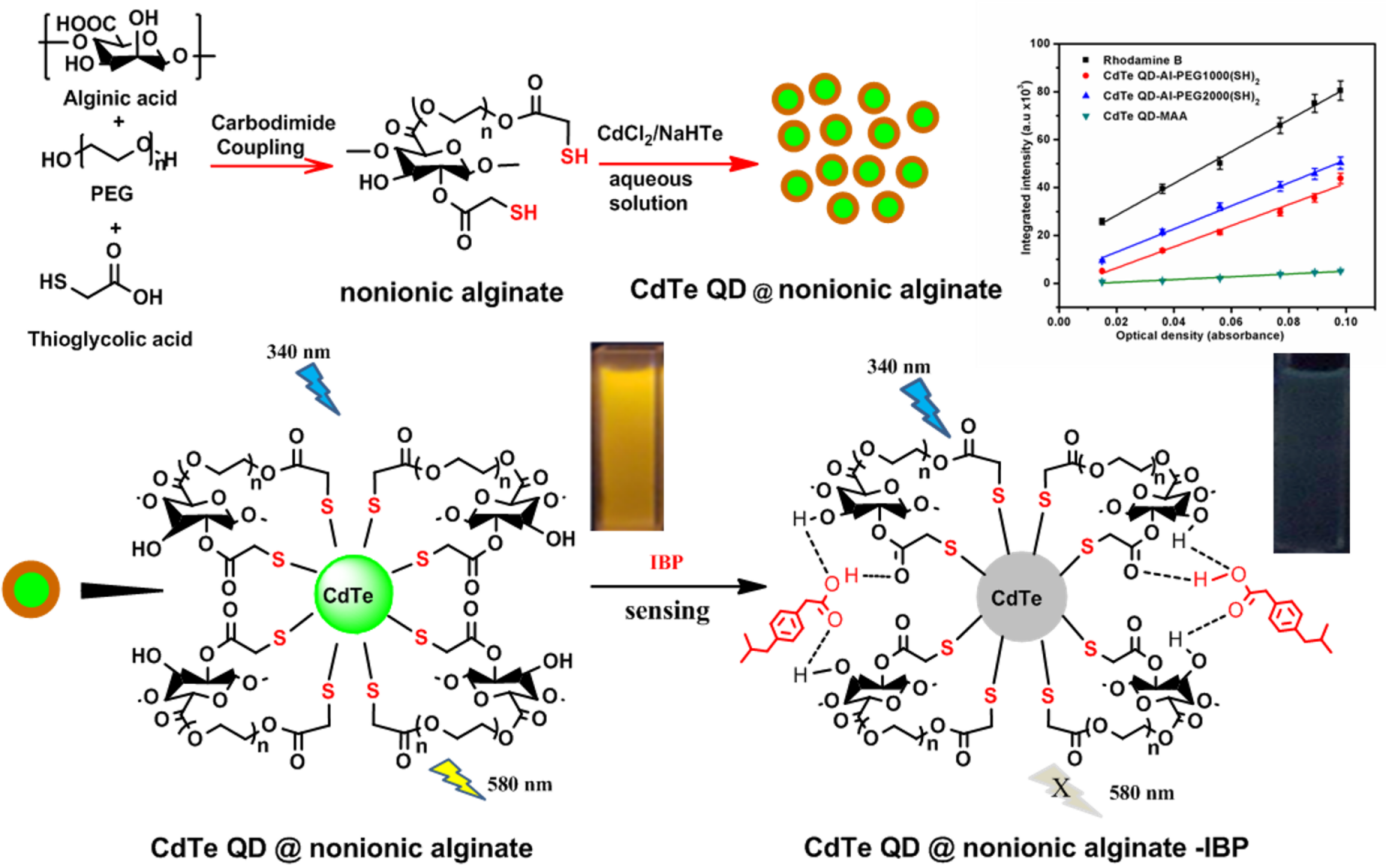
Synthetic route of CdTe QDs capped with nonionic alginate polymers for ibuprofen sensing. Figure 16 was reprinted from [11], Sensors and Actuators B: Chemical, Vol. 256, by S. M. Tawfik, B. T. Huy, M. Sharipov, A. Abd-Elaal, Y.-I. Lee, “Enhanced fluorescence of CdTe quantum dots capped with a novel nonionic alginate for selective optosensing of ibuprofen”, pages 243-250, Copyright (2018), with permission from Elsevier. This content is not subject to CC BY 4.0.

The recent developments regarding alginate-based nanoparticles with quantum dots (QDs) for pharmaceutical sensing applications hold great promise. For example, Tawfik et al. [11] used alginic acid and PEG with varying molecular weights (MW = 1000 and 2000 Da) to generate CdTe QDs in situ. The amphiphilic alginate shell in this system improved the fluorescence intensity, quantum yields, and biocompatibility of the CdTe QDs. Ibuprofen (IBP) quenched the fluorescence of QDs, which is explained by the hydrogen bonding interactions between IBP and QDs (Figure 16). A low LOD of 4 nM and a broad linear range of 1.0–30 μmol·L^−1^ were obtained. Furthermore, the sensor demonstrated good performance in practical applications along with good selectivity, repeatability, and stability.

**Figure 17:**
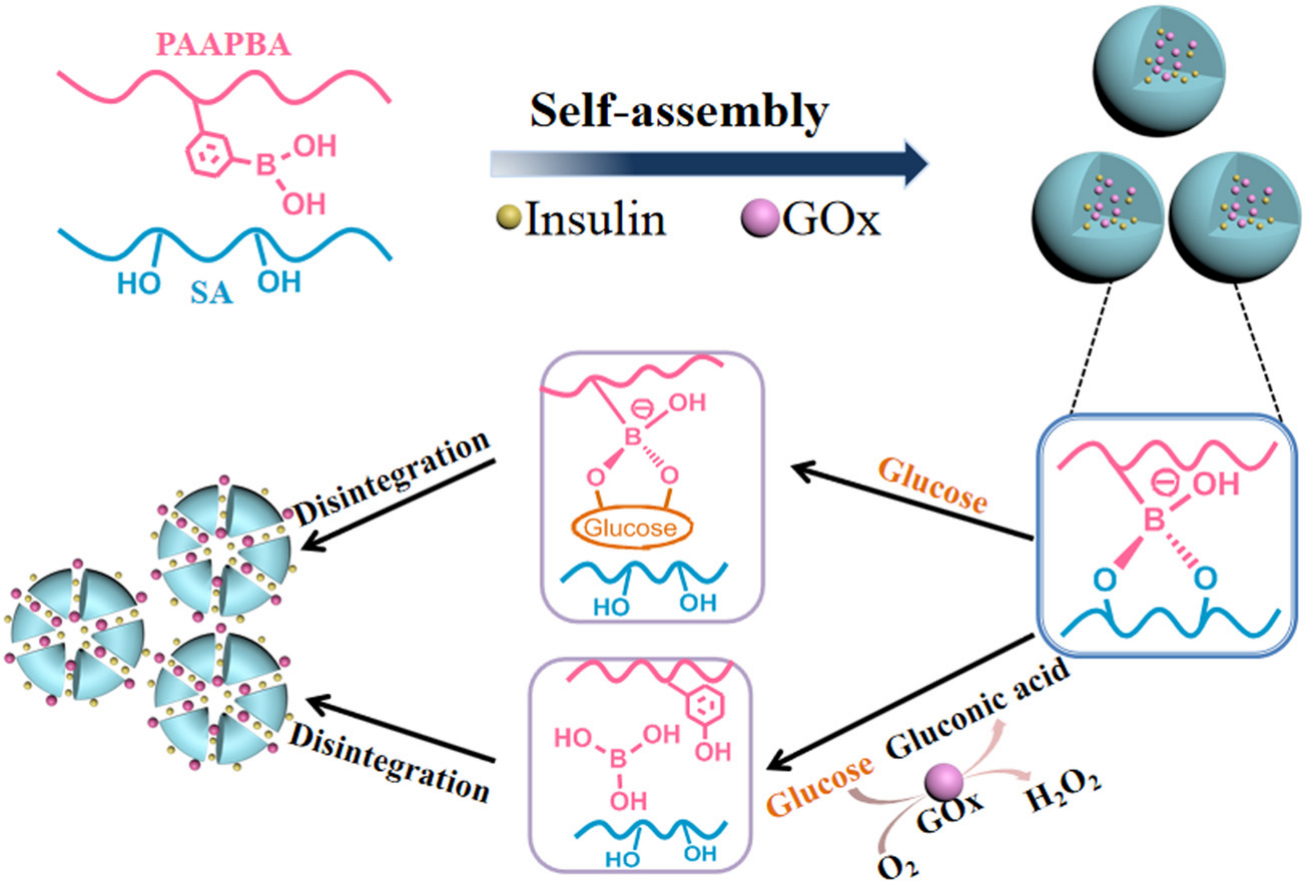
Fabrication of insulin/GOx-loaded NPs and mechanism for its response to glucose. Figure 17 was reprinted from [171], International Journal of Biological Macromolecules, Vol. 159, by Z. Chai, H. Dong, X. Sun, Y. Fan, Y. Wang, F. Huang, “Development of glucose oxidase-immobilized alginate nanoparticles for enhanced glucose-triggered insulin delivery in diabetic mice”, pages 640-647, Copyright (2020), with permission from Elsevier. This content is not subject to CC BY 4.0.

As researchers continue to explore and refine these nanoparticles, they have the potential to revolutionize pharmaceutical research, diagnostics, and drug monitoring, leading to personalized medicine and improved therapeutic outcomes. Alginate-based NPs have shown great promise in pharmaceutical drug delivery applications [171]. For example, glucose oxidase and insulin-containing alginate-poly(acrylamide phenylboronic acid) NPs were described as novel glucose-triggered insulin delivery system. These advanced biocompatible nanocarriers might have high therapeutic value in the treatment of diabetes because they are formed by the production of cycloborates and make use of the glucose/H_2_O_2_ dual responsiveness to enable a quicker release of insulin, as shown in Figure 17.

### Challenges and future directions

Alginate nanoparticles have emerged as promising carriers for various applications, including sensing and drug delivery. However, there are several challenges and limitations that need to be addressed to fully exploit the potential of alginate nanoparticles. One key challenge is the stability of alginate nanoparticles. Currently, alginate-based nanoparticles tend to have a wide size distribution and a partially charged surface due to their envelopment by cationic polymers. This leads to issues regarding their stability as well as their loading capacity and drug encapsulation efficiency. To overcome these limitations, researchers have been exploring different strategies. Some of these strategies include the use of ionic cross-linking with cationic polymers such as chitosan to improve the stability and loading capacity of alginate nanoparticles [172]. Another challenge is the scalability of alginate nanoparticle production. The current methods for producing alginate nanoparticles, such as ionic gelation, are complex and require precise control over factors such as solution viscosity and counter-ion concentration. This makes it difficult to replicate the resulting particles with a high degree of precision. To address this challenge, researchers are exploring alternative methods for producing alginate nanoparticles with better scalability and reproducibility. For instance, one approach is to use alginate and chitosan as surface modifiers in pre-formed nanoparticles made from a different polymer [173].

In addition to addressing the challenges and limitations, there are several future directions and areas of research in the field of alginate-based nanoparticles. These include developing methods for synthesizing size-monodisperse alginate nanoparticles with a fully negatively charged surface, improving the stability of alginate nanoparticles at room temperature, enhancing the loading capacity and drug encapsulation efficiency of alginate nanoparticles, and exploring alternative methods for scalable and reproducible production of alginate nanoparticles, such as microfluidics or electrostatic self-assembly. In addition, alginate nanoparticles have the potential for the use in biological cell imaging, acting as fluorescent probes for cell detection. Their hydrophilic character allows for increased loading of hydrophilic drugs, and they can modify the release profile of proteins and other macromolecules intended for oral administration when conjugated with dextran. Furthermore, alginate-stabilized semiconductor nanoparticles have the potential for applications in photocatalysis and can serve as adjuvants in vaccinations. Moreover, the use of sodium alginate in pharmaceutical applications has shown promising results in terms of drug delivery, particularly in the encapsulation of bioactive agents such as doxorubicin or paclitaxel for improved selectivity of drug release in cancer cells [174].

Overall, the future prospects of alginate-based nanoparticles in sensing and smart drug delivery are promising, with the potential for advancements in various pharmaceutical and biomedical applications such as targeted drug delivery, personalized medicine, biosensing, bioimaging, and diagnostics.

## Conclusion

The recent developments regarding alginate-based nanoparticles have shown great potential for targeted drug delivery and sensing applications. The unique properties of alginate, such as biocompatibility, biodegradability, and ability to encapsulate various therapeutic agents, make it an ideal candidate for several sensing applications. This review proved that alginate can be used to enhance the sensing ability of many sensors and can deliver them to the desired sensing locations. In addition, sensors using alginate have long-term stability. This review highlighted the recent sensing applications of alginate-based nanoparticles in environmental, biomedical, and pharmaceutical sensing. The versatile nature of alginate allows for the incorporation of various sensing elements, such as enzymes or antibodies, resulting in the highly sensitive and selective detection of target molecules. Moreover, advantages of using alginate-based nanoparticles in these applications were discussed. In addition to sensing, surface modifications of alginate nanoparticles enable tailored administration to specific cells or tissues, reducing off-target effects while increasing therapeutic efficiency.

However, challenges remain in optimizing the stability, scalability, release kinetics, and target specificity of alginate-based nanoparticles. Further research is needed to overcome these challenges and to translate the exciting developments into practical applications. With continued efforts, alginate-based nanoparticles have the potential to revolutionize targeted drug delivery and sensing, leading to improved therapeutic outcomes and diagnostic capabilities in various biomedical fields.

## Data Availability

Data sharing is not applicable as no new data was generated or analyzed in this study.
